# The Influence of Different Sustainable Silk-Based Fillers on the Thermal and Mechanical Properties of Polylactic Acid Composites

**DOI:** 10.3390/polym14225016

**Published:** 2022-11-18

**Authors:** José Miguel Ferri, Miguel Aldas, Emilio Rayon, Maria Dolores Samper, Antonio Abel Lozano-Pérez

**Affiliations:** 1 Instituto de Tecnología de Materiales (ITM), Universitat Politècnica de València (UPV), Plaza Ferrándiz y Carbonell 1, 03801 Alcoy, Alicante, Spain; 2 Departamento de Ciencia de Alimentos y Biotecnología, Facultad de Ingeniería Química y Agroindustria, Escuela Politécnica Nacional, Quito 170517, Ecuador; 3 Departamento de Biotecnología, Genómica y Mejora Vegetal, Instituto Murciano de Investigación y Desarrollo Agrario y Medioambiental (IMIDA), 30150 La Alberca, Murcia, Spain; 4 Instituto Murciano de Investigación Biosanitaria (IMIB)-Arrixaca; 30120 El Palmar, Murcia, Spain

**Keywords:** polylactic acid, composite, fillers, silk, fibers, microparticles, nanoparticles

## Abstract

In this work, different silk fillers combined with maleinized corn oil (MCO), as environmentally friendly plasticizers, were used to modify the mechanical and thermal properties of polylactic acid (PLA) composites. Melt extrusion and injection were used to obtain samples with a content of 10 wt.% of MCO and 0.5 phr of different silk fillers: crushed silk (CS), silk fibroin microparticles (SFM), and silk fibroin nanoparticles (SFN). PLA formulation with 10 wt.% of MCO and 0.5 g of CS per hundred grams of composite (phr) showed the highest increase in mechanical ductile properties with an increase in elongation at break of approximately 1400%, compared with PLA. Differential scanning calorimetry (DSC) showed a decrease of 2 °C in their glass transition temperature with the addition of different silk fillers. In addition, SFM and SFN increase the degree of crystallinity of PLA. This increment was also confirmed by infrared spectroscopy analysis. Field emission scanning electron microscopy (FESEM) images revealed a good dispersion of the different silk fillers. Among them, PLA formulation with 10 wt.% MCO and 0.5 phr of SFN, showed an optimal balance between maximum resistance and elongation at break, with 52.0 MPa and 10.8%, respectively, improving elongation at break by 635%. Furthermore, all samples were satisfactorily disintegrated under composting conditions.

## 1. Introduction

During 2021, approximately 2.4 million tons of bioplastics were produced, and nearly 1 million tons of bio-based and non-biodegradable polymers, mainly polyolefins, polyesters, and polyamides, which are usually manufactured by conventional processes, such as injection and extrusion, although for technical applications other transformation processes, such as pultrusion, can be used to obtain parts with improved characteristics [1,2,3]. In addition, there are more than 1.5 million tons of biodegradable bioplastics, such as polylactic acid (PLA), polybutylene adipate terephthalate (PBAT), and starch blends. According to the data compiled by European Bioplastics in cooperation with de Nova-Institute research institute, a 200% increase in the world production capacity is expected in the next 5 years, reaching more than 7.5 million tons of bioplastics in 2026 [4].

PLA is one of the most widely used biodegradable bioplastics, with 29% of global production [5]. In addition, there are different commercial grades available for various applications, and it is approved by the U.S. Food and Drug Administration (FDA) as a safe polymer for food contact [6]. At the same time, it can be easily processed with traditional equipment for the transformation of plastic materials of petrochemical origin, and the PLA-based formulations can be disintegrated under composting conditions [7,8,9]. On the other hand, under natural conditions, it does not decompose easily and can be used for components with a long service life [10]. However, its sensitivity to moisture, susceptibility to aging, high stiffness, and low elongation at break lead to the modification of PLA.

To modify the properties of PLA, different actions can be carried out. On the one hand, it can be mixed with other polymers that have greater ductility and good compatibility to reduce stiffness [11,12,13]. Plasticizers can also be used; they can be of petrochemical origin, such as poly(ethylene glycol), but it is better if they are plasticizers of natural origin, such as vegetable oils [14,15]. On the other hand, incorporate different fillers, from organic, inorganic, macro, micro, or nano-fillers [10,16,17,18]. Modified vegetable oils and lipid derivatives have been extensively studied as plasticizers to improve the ductility of PLA, reaching an improvement of 200% incorporating 5 wt.%.

The incorporation of different derivatives of vegetable oils, such as epoxidized karanja oil [8], epoxidized palm oil [19], maleinized linseed oil (MLO) [20], epoxidized and maleinized cottonseed oil [21,22], acrylate epoxidized soybean oil (AESO) [23], and monomethyl itaconate epoxidized downstream corn oil [24], has improved the toughness of PLA. This improvement is explained by possible hydrogen bonds between the PLA and the reactive groups of the oils [14].

Polymer nanocomposites are of great practical interest and PLA-based polymeric materials have become extremely attractive materials for a wide range of applications. In recent years, the addition of organic fillers coming from renewable resources has been presented as an interesting method for improvement of the properties of polymers, such as poly-Lactic acid (PLA), availability due to the renewable origin, low cost, and biodegradability among others. Introducing small solid filler particles in a polymer matrix significantly increases the elastic modulus, wear resistance, and damping properties. However, the reinforcing effect depends not only on the filler amount but also on filler surface chemistry, compatibility between the filler and the matrix, filler structure, size, and filler distribution [25].

If a filler drives the mechanical properties, important parameters are therefore filler composition, size, morphology, and secondary structure in the sample. However, it is experimentally difficult to obtain well-controlled filler morphology and distribution state to understand their effect on material behavior.

Silk fibroin (SF) has been used in several technological applications due to its high mechanical strength, although recently other interesting properties, such as biodegradability, are being pointed out. Recent studies have shown the possibilities of using SF biomaterials as organic fillers for PLA or PLA/PCL composites, improving their mechanical properties and showing good composting biodegradability [16,26,27].

SF is the structural protein of the silk produced by the silkworm *Bombyx mori* (*B. mori*), and this unique material has historically been highly valued for its strength and luster [28]. In terms of sustainability, due to the large-scale cultivation of silkworms for the textile industry, there are abundant and reasonable cost sources for this natural polymer and the possibility of using SF in a wide variety of formats, such as films, scaffolds, hydrogels, and micro- or nanoparticles for drug delivery, has led to many interesting applications [29].

Native *B. mori* silk is composed of a pair of SF filaments coated and glued by sericin proteins. The SF consists of a light chain (Mw ~26 kDa) and a heavy chain (Mw ~390 kDa) linked by a disulfide bond, and their outstanding properties arise from their combination of secondary structures. SF is a block copolymer rich in hydrophobic *β*-sheet-forming blocks linked by small hydrophilic linker segments or spacers [30]. This combination of crystalline regions and amorphous and flexible domains results in a hydrophobic protein that self-assembles to form strong and resilient materials. The dominance of the *β*-sheet-forming regimes within the fibroin structure imparts the protein-based materials with high mechanical strength and toughness.

In terms of strength, the ultimate tensile strength of *B. mori* silk fibers is 740 MPa [31], silkworm silk is superior to commonly used polymeric degradable biomaterials, such as collagen and poly-(l)-lactic acid (PLA).

In addition, the degradation rate of silk can be altered by the mode of processing the fibroin, as well as post-processing treatments, related to the content of *β*-sheet crystals and the degree of organization of the non-crystalline domains. In general, the degradation rate decreases with an increase in overall *β*-sheet content [29].

Thus, the use of environmentally friendly plasticizers, such as maleinized corn oil (MCO), and different silk fillers were successfully mixed with poly(lactic acid) (PLA) to modify their mechanical and thermal properties, increasing their potential as sustainable and compostable biocomposite.

## 2. Materials and Methods

### 2.1. Materials

PLA Ingeo^TM^ Biopolymer 2003D was supplied in pellets form by NatureWorks LLC (Minnetonka, MN, USA). This PLA grade has a melt flow rate (MFR) of 0.6 g·min^−1^ measured at 210 °C and 2.16 kg and a true density of 1.24 g·cm^−3^. Maleinized corn oil (MCO) was obtained through the modification of food-grade commercial corn oil (COOSOL, Vilches, Spain) using maleic anhydride (MA). MA was 98% pure and was supplied by Sigma-Aldrich (MERCK, Darmstadt, Germany). The corn oil maleinization process was carried out in a three-necked round-bottom flask with a capacity of 500 mL to be able to incorporate all the necessary elements. One of them was used to introduce the reagents, another to control the temperature and the central one to connect the reflux condenser. The maleinization ratio used was 2.4:1, following the recommendations of previous works [21,32,33]. A total of 81 g of MA and 300 g of corn oil were used, the epoxidation process was carried out in three stages at different temperatures, 180, 200, and 220 °C, and samples were taken to calculate the acid value (AV) every 60 min.

**Figure 1:**
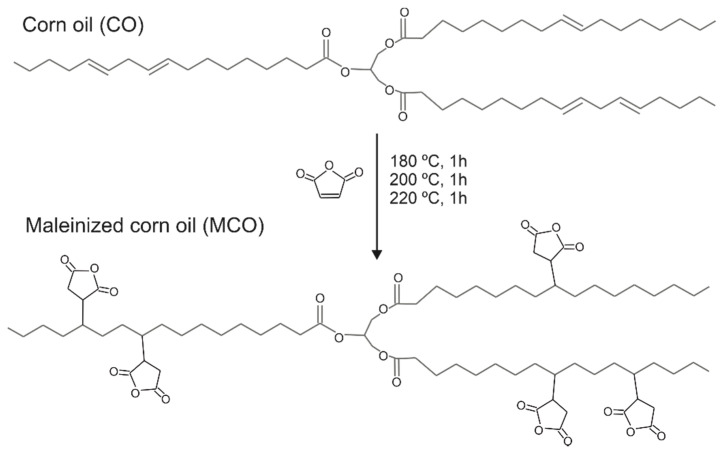
Schematic representation of the maleinization process of corn oil.

Initially, the oil was introduced into the round flask and when it reached the temperature of 180 °C, 27 g of MA (1/3 of the total) were added. This temperature was maintained for one hour and increased to 200 °C, then additional 27 g of MA were added and the mixture was maintained again for 1 h and the same process was carried out at 220 °C. Finally, the mixture was left to cool down to room temperature. The degree of maleinization was analyzed utilizing the acid value, it was verified in each step during the maleinization process, and the acid value increased from 0.15 mg KOH g^−1^, before starting the reaction process, to 109.2 mg KOH g^−1^ at the end of the maleinization process, which indicates that the maleinization process has been carried out correctly (see Supplementary Material Figure S1). A schematic representation of the maleinization process can be seen in Figure 1.

White silk cocoons used in the experiments (see Supplementary Material Figure S2) were obtained from *B. mori* silkworms fed with fresh *Morus alba L.* leaves and were reared in the sericulture facilities of the Instituto Murciano de Investigación y Desarrollo Agrario y Medioambiental (IMIDA, Murcia, Spain). The intact chrysalides were extracted manually from the cocoons before silk processing.

Crushed silk (CS) microfibers were obtained by direct milling of the cocoons by using an IKA MF-10 basic microfine grinder (IKA^®^-Werke GmbH & Co., KG, Staufen, Germany) equipped with a stainless-steel sieve of Ø 1.0 mm (IKA Ident. No.: 0002939200). The cotton-like powder was collected in a plastic recipient, sealed, and stored at room temperature until use (see Supplementary Material Figure S3).

SF micro (SFM) and nanoparticles (SFN) were prepared by using an SF aqueous solution following the procedure described by Rodriguez-Nogales et al., with slight modifications [34]. Briefly, the silk cocoons were degummed in a solution of Na_2_CO_3_ 0.05 M for 120 min, to remove sericin and other impurities [35]. Then, purified SF was dissolved in Ajisawa’s solvent system composed of a mixture of CaCl_2_/EtOH/H_2_O, at a 1:2:8 molar ratio [36] and dialyzed against distilled water for 3 days in a Snakeskin Dialysis Tubing 3.5 KDa MWCO (Thermo Fisher Scientific, Waltham, MA, USA) with eight total water changes. Finally, the obtained SF aqueous solution was filtered to remove small impurities and aggregates. Then, two aliquots of the SF solution were processed separately.

On one hand, SF aqueous solution was injected in a Büchi B-290 spray-dryer (BÜCHI Labortechnik AG, Switzerland), as described by Rodriguez-Nogales et al. [35]. The inlet temperature was set at 120 °C, the pump flow rate was set to 20% (6–7 mL·min^−1^), and the atomizing air flow rate was at 600 L·h^−1^ (0.75 bar). The microparticles were obtained as a light white powder (see Supplementary Material Figure S3). On the other hand, the second aliquot of the SF aqueous solution was dropped into methanol (at a final ratio of methanol/aqueous silk 9:1) to form the SFN by rapid desolvation as previously described [34]. Then, SFN were recovered by centrifugation, washed with water (3×), and freeze-dried to powder (see Supplementary Material Figure S3).

All other chemicals and solvents were purchased from Merck (MERCK, Darmstadt, Germany) and were of analytical grade and used without further purification unless otherwise specified.

**Figure 2:**
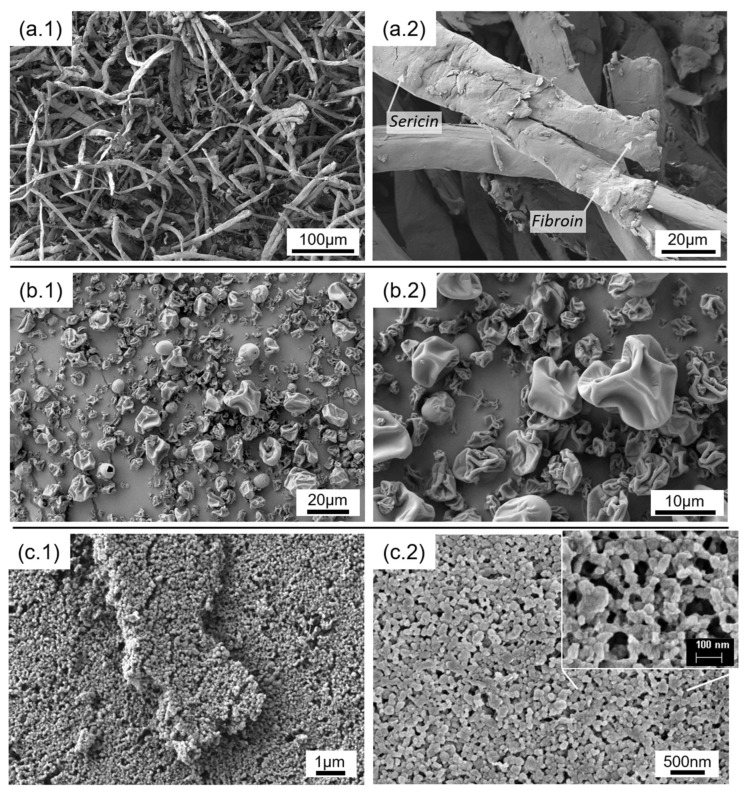
FESEM images of the prepared silk fillers: crushed silk microfibers (**a.1**,**a.2**), sprayed SF microparticles (**b.1**,**b.2**), and SF nanoparticles (**c.1**,**c.2**) at different magnifications.

The prepared silk fillers were observed by electron microscopy. Figure 2 shows the FESEM images of the different silk fillers before mixing with MCO, captured under two magnifications. The CS was revealed as flattened silk fibers between 15 and 24 microns thick. At higher magnifications, the thin sericin layer is displayed enveloping a pair of fibroin fibers. The silk powder prepared by spray drying (SFM) was revealed as spherical particles in the range of 2 to 15 µm in diameter. The walls of the hollow microspheres collapse (implode), probably due to the generated stresses during the water evaporation process. Regarding the SFN, the FESEM images reveal a material formed by spherical nanoparticles in the range of 50 to 100 nm in diameter. In both cases, the morphology agrees with the previously reported for these particles [34].

### 2.2. Sample Preparation

Before processing, the PLA pellets were dried at 70 °C for 24 h to eliminate the moisture contained. The necessary amount of silk fillers needed to reach a final concentration of 0.5 phr in the composite, were first mixed with the MCO (ratio 9:1) to improve the dispersion of the filler in the PLA. The mixture of the different silk biomaterials and MCO was assisted by using the ultrasound technique in a Sonoplus HD 2200 equipment (Bandelin Electronic GmbH & Co., KG., Berlin, Germany) at an amplitude of 33% for 5 min. As MCO at room temperature is a highly viscous oil, it was previously heated to 50 °C and during ultrasonic-assisted dispersion, the temperature increased to 120 °C due to internal friction. The content of the silk-based materials, MCO and PLA, and the code used can be seen in Table 1.

**Figure 3:**
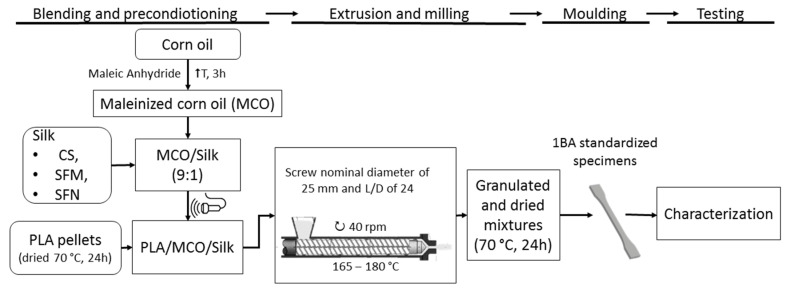
Flow chart of the process used for the preparation of PLA-MCO-Silk composites.

Once the different homogenized silk-MCO mixtures were obtained, they were added to the PLA pellets and mixed manually in a ZIP bag, and later they were introduced in a twin-screw co-rotating extruder (DUPRA S.L., Alicante, Spain) with a screw nominal diameter of 25 mm and L/D of 24. The setting temperatures along the extruder (from feed zone to die) were 165, 170, 175, and 180 °C, respectively. The screw speed was kept at 40 rpm. After extrusion, each material was cooled at room temperature, granulated, and dried at 70 °C for 24 h. The pelletized materials were injection molded into 1BA standardized specimens for tensile tests following the ISO 527-1:2019 [37] and rectangular specimens (80 × 40 × 4 mm^3^) for flexural and Charpy impact test [38] with an injection molding machine Sprinter-11 (Erinca S.L., Santa Coloma de Cervello, Spain). The injection molding was carried out with a temperature profile of 170, 175, and 180 °C from the hopper to the nozzle, respectively. For clarity, the flow chart of the process is presented in Figure 3.

Previously to the UV-visible measurements and disintegration under composting conditions analyses, all formulations were processed into films in a 10-Tn hydraulic press (Robima S.A., Valencia, Spain) through a compression molding process developed at 180 °C. The press was equipped with two hot aluminum plates and a temperature controller (Dupra S.A., Castalla, Spain). A 120 × 120 mm^2^ film mold was used to transform the pellets of each formulation into films. The pellets were kept between the plates at atmospheric pressure for 4 min until melting and further submitted to the following cycle: 1 min at 10 MPa pressure, 2 min at 25 MPa pressure, 1 min at 50 MPa, and finally quenched to room temperature at atmospheric pressure during 4 min. Squared films of 400 µm of thickness were obtained.

### 2.3. Characterization

Samples were studied and characterized to determine not only the mechanical and thermal properties but also the structural and spectroscopic characteristics.

#### 2.3.1. Mechanical Properties

The tensile and flexural mechanical properties were determined using a universal test machine Ibertest ELIB 30 (S.A.E. Ibertest, Madrid, Spain) following the ISO 527 [39] and ISO 178 [40] standards respectively. The tests were carried out with a 5 kN load cell and a crosshead speed of 5 mm·min^−1^ for both tests. At least five specimens of each sample were tested at room temperature and the averaged results were shown for each prepared sample. Impact testing was performed following ISO 179 [41] standard with a Charpy impact pendulum (Metrotec S.A., San Sebastian, Spain) with a 6 J pendulum, and five different samples for each formulation were tested. Significance in the mechanical data differences was statistically analyzed under the same conditions specified for data treatment, that is, using the one-way analysis of variance (ANOVA), through OriginPro 2018 software, at 95%.

The elastic modulus (E) and hardness (H) of each prepared PLA formulation were measured using a G200 Nanoindenter (Agilent Nanotech, Santa Clara, CA, USA). A matrix of 25 indentations was tested in the cross-section of each sample at 2000 nm constant depth and a strain rate of 0.05 s^−1^. The H and E values were calculated using the Continuous Stiffness Measurement method over a depth range of 1000–1500 nm. Samples were prepared by cryogenic temperature fracturing and then polished by a common metallographic preparation process.

#### 2.3.2. Thermal Properties

The thermal stability of the different formulations processed was analyzed by thermogravimetric analysis (TGA) using a TGA/SDTA 851 thermobalance from Mettler-Toledo Inc (Schwerzenbach, Switzerland). Samples with a total weight between 10–15 mg were heated at a rate of 10 °C·min^−1^ from 25 to 800 °C under a nitrogen atmosphere (gas flow rate 50 mL·min^−1^). The temperature at the degradation starts was determined at 5 % mass loss (T_5%_), while the maximum degradation temperature (T_max_) of each of the stages was calculated from the peak of the first derivative of the TGA curves (DTG).

The thermal behavior of the different formulations was analyzed using a Mettler-Toledo 821 Differential Scanning Calorimetric (DSC) (Mettler-Toledo Inc., Schwerzenbach, Switzerland) under a nitrogen atmosphere (flow rate 66 mL·min^−1^). Samples weighing approximately 7 mg were placed in the aluminum pans and heated from −50 to 220 °C with a heating rate of 10 °C min^−1^. The degree of crystallinity of PLA (*Χ_c_*_,PLA_) was calculated by equation 1,

(1) $$X_{c\;}\left(\%\right)=100\times \left[\frac{\Delta H_{m}}{\Delta H_{0}\cdot w}\right]$$

where Δ*H_m_* (J·g^−1^) is the thermodynamic melting enthalpy per gram, Δ*H*_0_ (J·g^−1^) is the theoretical melting enthalpy associated with the 100% crystalline polymer, which in the case of PLA (Δ*H*_0,PLA_) is 93 J·g^−1^ [22], and *w* (g) is the weight fraction of PLA.

#### 2.3.3. Dynamic Mechanical Thermal Analysis (DMTA)

Dynamic mechanical thermal analysis (DMTA) was used to measure the property of the evolution of the storage modulus (G’) and the loss factor (*tan δ*) concerning the temperature of the different formulations. An AR G2 oscillatory rheometer (TA Instruments, New Castle, DE, USA) was used in rectangular torsion mode. Rectangular samples with dimensions of 40 × 10 × 4 mm^3^ were analyzed at 1 Hz in the temperature range of 30 to 150 °C with a heating rate of 2 °C·min^−1^ and a maximum shear strain (γ) of 0.1%. The glass transition temperature (T_g_) was determined as the position of the maximum peak values of *tan δ*.

#### 2.3.4. Spectroscopic Analysis

On one hand, attenuated total reflectance Fourier-transform infrared spectroscopy (ATR-FTIR) was used to analyze the secondary structure of samples. Each spectrum was acquired using a Nicolet iS5 spectrometer equipped with an iD5 ATR accessory (Nicolet, Thermo Scientific, Waltham, MA, USA) and controlled with OMNIC v.9.7. software. Measurements were performed in absorbance mode with a resolution of 4 cm^−1^, a spectral range of 4000–550 cm^−1^, and 64 scans, using N-B strong apodization and Mertz Phase correction. The analysis was finally focused on the range of 1700–1100 cm^−1^ as the most informative for the IR spectra of silk proteins.

On the other hand, UV-visible measurements were made over 400 µm thickness films using a spectrophotometer Cary 100 UV–Vis (Agilent technologies, Barcelona, Spain). The UV–Vis absorption spectra were recorded in the range of 200–800 nm to verify the optical differences among the formulations.

#### 2.3.5. Microscopic Characterization

The silk fillers and the microstructure of the cross-sections of fractured composites were observed by a field emission scanning electron microscope (FESEM), ZEISS ULTRA 55 (Oxford Instruments, Pleasanton, CA, USA). All samples were sputtered with a thin layer of platinum, using a Leica Microsystems (Buffalo Grove, IL, USA) EM MED0200 high vacuum sputter coater and observed by applying an accelerating voltage of 2 kV and using a secondary electrons detector.

#### 2.3.6. Static Water Contact Angle Measurements

The water contact angle (*θ*_C_) was measured on the surface of injection samples at room temperature using an Easy Drop Standard goniometer FM140 (KRÜSS GmbH, Hamburg, Germany). The equipment was provided with a camera and analyzer software (Drop Shape Analysis SW21; DSA1 from KRÜSS GmbH, Hamburg, Germany). Five contact angles were measured randomly using distiller water as contact liquid on the surface film with a micro syringe.

#### 2.3.7. Disintegration under Composting Conditions

Disintegration tests were conducted on 25 × 25 mm^2^ squared films with an average thickness of 400 µm under the recommendations of the ISO 20200:2015 standard [42]. Samples were placed into an aerobic reactor with synthetic compost waste manufactured as indicated in ISO 20200:2015. Before placing samples into the reactor, all films were dried at 40 °C for 24 h. Then, the films were buried into the compost soil and extracted at 1, 7, 14, 21, 30, 45, and 60 days, washed with distilled water and dried at 40 °C for a day, and reweighted. The disintegration degree of each formulation was calculated by normalizing the recovered sample weight at each time to the initial value. Finally, the ATR-FTIR spectra of the samples were recorded, as previously detailed, to identify the changes in the secondary structure of the materials along the disintegration process.

## 3. Results and Discussion

### 3.1. Mechanical Properties

The mechanical properties of the prepared PLA formulations were acquired by employing tensile and flexural stress, Charpy’s impact energy, and the nanoindentation technique. Table 2 resumes the tensile, flexural, and impact resistance properties. The neat PLA showed the maximum stress-resistance value, obtaining 62 and 102 MPa for tensile and flexural stress, respectively. Nevertheless, these values were significantly reduced when the MCO was added, as expected by their plasticizing behavior. These results are in agreement with previous reports when working with PLA and other modified oils [8,9]. While the MCO oil decreases the strength, it achieves a considerable increase in the elongation at break, reaching 1.7% for PLA, regarding the 6.1% by MCO. Only the impact resistance slightly decreases.

The addition of different silk particles resulted in a reduction of their maximum resistance while elongation was notably increased, achieving 23.7% for the CS fibroin additive. The resistance under impact also was increased for the CS and SFM additives (31 kJ·m^−2^), overcoming the neat PLA impact energy value of 29.5 kJ·m^−2^. These results lead us to think that fibroin additives are working as plasticizers, decreasing the stiffness while elongation and impact resistance is notably increased. The differences observed between additives are initially understood based on the different chemistry of the additives: the CS additive contains sericin, while the micro and nanoparticulated do not, and the fact of the additives could be stiffer than the matrix phase, leading it to work as reinforcing fillers. Depending on the balance obtained between the plasticizing and reinforcing effect, different results would be obtained. In this sense, it was required to have information about phase coherence, additive surface area, and the nature of the relationship between matrix and additives. For this reason, the stiffness of the matrix phase was also analyzed by nanoindentation technique, and the microstructure of the samples was observed by electron microscopy.

**Figure 4:**
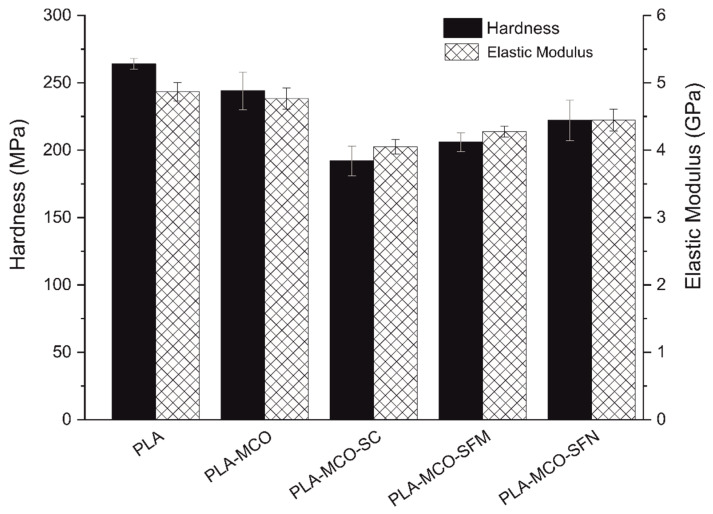
Hardness and elastic modulus acquired for each formulation by nanoindentation technique.

Thus, Figure 4 resumes the hardness and elastic modulus of samples obtained by nanoindentation. Indentations performed under very low loads permit us to acquire the stiffness properties of the matrix without the possible reinforcement effect of the micro additives that are affecting under tensile or flexural stress tests, this is not applicable for SFN that probably would be in high content inside the indented material volume. Nanoindentation results corroborated the stiffness loss when MCO is added, diminishing from H = 260 MPa to 250 MPA and E = 4.8 GPa to 4.7 GPa, for the neat PLA and PLA-MCO, respectively. These results agree with previously reported values obtained by this technique [43]. One of the most significant results is that the whole silk additives proved in this study reduced the hardness and elastic modulus below the PLA-MCO value and there is a stiffness increase tendency of CS, SFM, and SFN formulations as can be seen in Figure 3. Thus, silk fillers could be acting as a plasticizer, and the lowest value obtained by the CS formulation led us to think that the presence of sericin, which is present in this additive formulation, could also act as a second plasticizer substance. The higher H and E values obtained for SFN additive could be explained by the reinforcement behavior of the nanoparticles which block the elastic and plastic deformation inside low volumes of material [44]. These results show that the filler morphology of the nanoparticles compensated for the loss of stiffness caused by the addition of fibroin to the matrix phase, reaching values close to the PLA-MCO formulation.

### 3.2. Thermal Properties

The properties, and hence the applications, of the hybrid PLA-based plastics, depend greatly on the crystalline structures and the interaction among the components in the polymer, which will define their thermal properties. The effects of the different organic fillers on the thermal behavior of PLA, studied using differential scanning calorimetry (DSC) and thermogravimetric analysis (TGA), are presented here. Table 3 compiles the main thermal properties obtained by DSC and TGA. As can be observed, MCO modified the thermal properties of PLA except in glass transition temperature (T_g_).

Neat PLA has a T_g_ located at 62.4 °C, similar to the PLA-MCO formulation (62.3 °C). However, the incorporation of the different silk particles showed some effect on T_g_, being up to 2 °C lower than for neat PLA. In general, a decrease in the cold crystallization temperature (T_cc_) between 4 °C to 6 °C is observed for all formulations containing MCO, concerning neat PLA. On the other hand, an increase of 2 °C to 4 °C in the melting temperature (T_m_) is observed. An increase in molecular motion due to the plasticizing effect of the MCO is responsible for the increase of the degree of crystallinity (*Χ*) (even though it is small) and consequently the slight increase in T_m_. It should be noted that this grade of PLA is very amorphous and usually has low *Χ*. It is observed that PLA without MCO has a *Χ* of 0.6%, whereas, as MCO is added, the *Χ* increases up 1.7%. When the different types of silk particles are added, the *Χ* increases, although this increase is insignificant, reaching 3.9% for the PLA-MCO-SFM composite. A higher increase was reported by Carbonell-Verdú et al. [21], who demonstrated that by adding 10 phr of commercial maleinized linseed oil (MLO) and maleinized cotton seed oil (MCSO) to PLA, the increase of *Χ* was up to 11.6% and 19.1%, respectively.

The thermal stability of neat PLA and PLA-Silk composites was also evaluated using thermogravimetric analysis (TGA). Table 3 also shows some parameters related to thermal degradation. In particular, the temperatures at which a 5% weight loss occurs (T_5%_) and the T_max_ are summarized. In terms of thermal stability, it is observed that neat PLA and PLA-Silk composites show marked changes. Neat PLA presents a T_5%_ of 305.0 °C and a T_max_ of 339.6 °C. By incorporating MCO on PLA, a significant increase in stability is achieved as already observed in the work of Ferri et al. where different contents of maleinized linseed oil (MLO) were added to the PLA-30TPS mixture [45]. In the present work, the addition of 10 wt.% of MCO results in an increase of 21.3 °C in T_5%_ and 18.7 °C in T_max_. The thermal stability of the PLA-Silk composites is higher in all cases (between 3 °C and 4 °C in T_max_), demonstrating the good compatibility and interaction between PLA and silk particles. Similar behavior was observed by Deng et al. for poly(*d*,*l*-lactic acid)/silk fibroin (PDLLA/SF) composites [46].

### 3.3. Dynamic Mechanical Thermal Analysis (DMTA)

**Figure 5:**
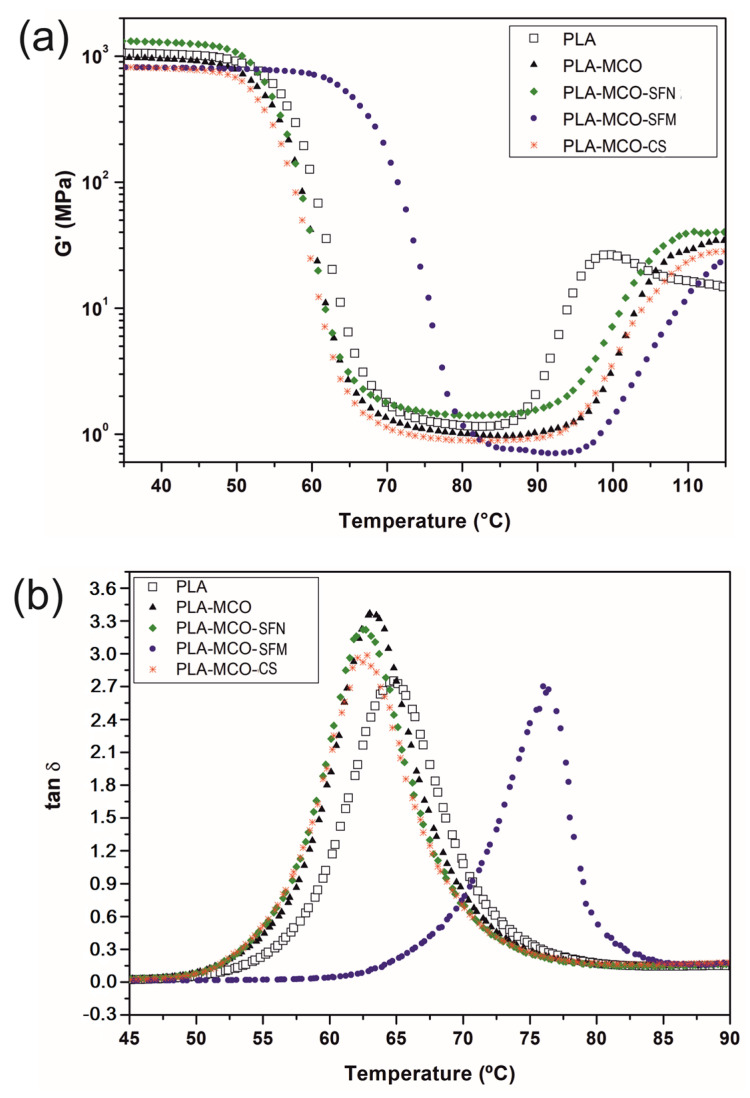
Effect of the temperature on the storage modulus (G′) (**a**) and damping factor (*tan δ*) (**b**) of neat PLA, plasticized PLA, and PLA-Silk formulations.

The viscoelastic behavior of PLA-Silk composites presented two distinctive changes in the storage modulus, as is shown in Figure 5. The first one, between 50 °C to 80 °C, is the drop of storage modulus (G′) which is related to T_g_ above 60 °C and the second one, between 90 °C to 110 °C, is identified as the T_cc_. The addition of MCO to PLA resulted in a loss of G′ at lower temperatures since it acts as a plasticizer, which provides greater free volume between PLA chains, decreasing the interaction between them [47]. While neat PLA reveals a G′ value of 1041 MPa while the plasticized PLA formulation (PLA-MCO) showed a decrease up to 948.8 MPa due to the plasticization effect. In addition, a delay in the onset temperature of T_cc_ was observed, meaning that crystallization occurs at higher temperatures. The onset T_cc_ temperature increased from 85.7 °C (neat PLA) to 93.8 °C for PLA-MCO formulation. This is due to the steric impediment exerted by the MCO on the PLA chains. The addition of the three types of silk-derived loads showed three distinct effects on PLA composites. The composite with CS shows a significant loss of G′ to 801.5 MPa at room temperature. The SFM also showed the same effect, since the value of G′ = 810.3 MPa is similar to that of the sample on the PLA-MCO-CS. However, SFN increased the stiffness of the PLA-MCO-SFN composite, which showed a G′ = 1289 MPa, even compared to the stiffness of neat PLA.

On the other hand, different effects on the T_g_ related to the damping factor (*tan δ*) peak of the composites are also observed in Figure 5b, depending on the type of silk material added. All formulations containing MCO except that containing microparticle (PLA-MCO-SFM) show a lower T_g_ than that of neat PLA. Specifically, the T_g_ values are reduced from 64.8 °C (neat PLA) up to 63.0 °C, 62.8 °C and 62.5 °C in PLA-MCO, PLA-MCO-CS, and PLA-MCO-SFN, respectively. This tendency is according to Perez-Nakai et al. who observed that increasing the content of maleinized Brazil nut seed oil (MBNO) or maleinized Hemp seed oil (MHO) plasticizers in PLA led to a decrease in the temperature of *tan δ* peak [32].

However, the PLA-MCO-SFM composite shows their *tan δ* peak at 76 °C, which was more than 10 °C higher than that shown by neat PLA. This behavior can be explained by the generation of hydrogen bonds among the hydrogens of the amide groups (R-NH) of the partly amorphous fibroin chains and the carbonyl groups (C=O) of the PLA chains, as described by Deng et al. [46]. Therefore, the increase in the interactions is equivalent to an increase in the apparent molecular weight of the PLA chains, which consequently need a higher supply of energy to be moved and the *tan δ* peak appears.

### 3.4. Spectroscopic Analysis

**Figure 6:**
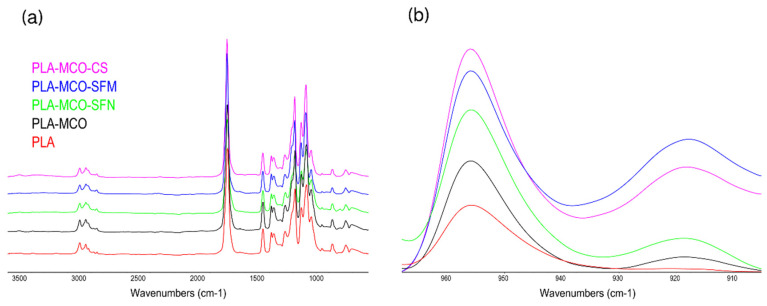
ATR-FTIR spectra of the Silk-MCO-PLA composites. (**a**) Full spectra of the composites. (**b**) Selected region of the spectra used to measure the relative crystallinity of PLA.

Previous studies have shown that IR spectroscopy is sensitive to the aggregated structure of PLA and PLA-Silk composites [27,48]. The degree of crystallinity is one of the most important properties of a semi-crystalline polymer, and it influences many of their mechanical properties, including modulus, hardness, and stiffness [48]. In this regard, ATR-FTIR analysis of the samples revealed that all the full spectra of the samples showed a similar profile, as can be seen in Figure 6a, but when those are observed in detail, clear differences arise in the enlarged spectral region of 1000–800 cm^−1^, with bands at approximately 955 and 921 cm^−1^, (Figure 6b). Thus, the band at approximately 921 cm^−1^ was assigned to the characteristic bands of 10_3_ helical crystalline conformations and the band at 955 cm^−1^ to the amorphous phase, and therefore the intensity ratio of 921 and 955 cm^−1^ can be used to measure the relative crystallinity of PLA [48,49].

As observed, the prepared PLA sample presented a predominantly amorphous structure and the addition of MCO and silk fillers increased the crystallinity of the structure. The sample containing silk microparticles reached the highest crystallinity among the measured samples, and this corroborates the observed results of the thermal properties of PLA-MCO-SFM.

**Figure 7:**
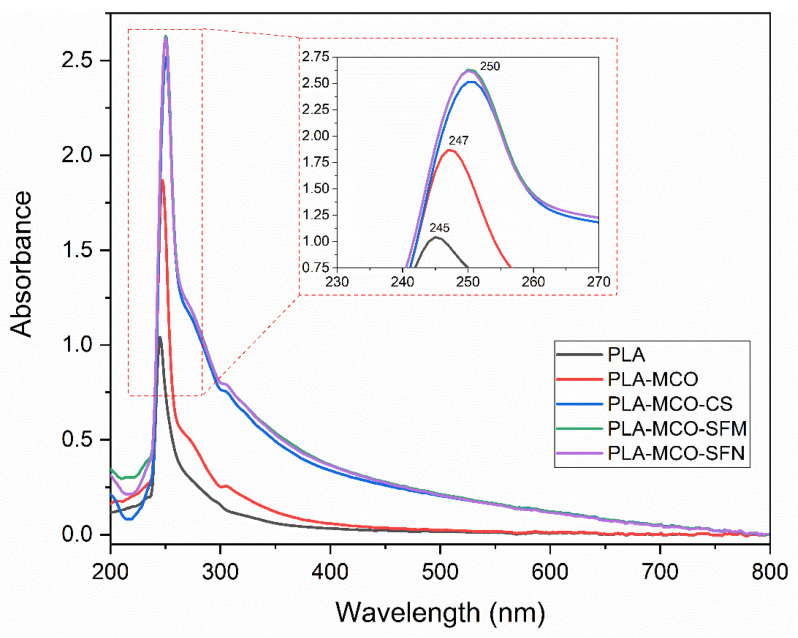
UV-vis absorbance spectra of PLA and PLA blended with MCO and silk, and the expanded area between 230 and 270 nm.

On the other hand, all the UV-visible spectra have the same pattern as neat PLA. As expected, no new absorption bands appeared. The UV absorption spectra of the samples are shown in Figure 7. The curves present a maximum absorption peak in the UV zone, 240 and 270. (PLA maximum (245 nm)). However, the addition of MCO and silk produced a bathochromic effect—the maximum peak is shifted to higher wavelengths (lower frequencies)—as can be seen in the expanded area of Figure 7. This phenomenon suggests either an interaction between these materials [50], and/or a shift due to the dispersion and size of the added particles [51], and the intrinsic absorbance of the SFs in the lower region of the spectrum (at λ = 280 nm) provided by the presence of aromatic residues, such as Tyr (5.4 wt.%) or Phe (1.6 wt.%) [52].

It can be observed that MCO increased the absorbance in the maximum absorption peak of PLA (UV region). MCO and silk do not affect the color change of films (400–700 nm). However, the combination of both increases the absorbance of the samples in all the UV-Vis range, which suggest a reduction in the film transparency [53]. This could be because silk particles act as physical obstacles that interact with light and prevent light from transmitting through the samples [54]. However, this absorbance increment provides a blocking effect in the UV region (240 to 400 nm) and the potential to UV-shield and highly preserve the intrinsic optical property of the substrate onto which it may be mounted. The principal chromophores absorbing in the UV region are considered to be the aromatic amino acids, tyrosine, phenylalanine, and tryptophan, which are present in the silk structure [55]. In addition, the difference between silk particle size in the absorption pattern of the samples is marginal. Therefore, based on the UV absorption results, silk can be used in any of the studied presentations (CS, SFM, or SFN) as a UV light shield in PLA.

### 3.5. Microscopic Characterization

**Figure 8:**
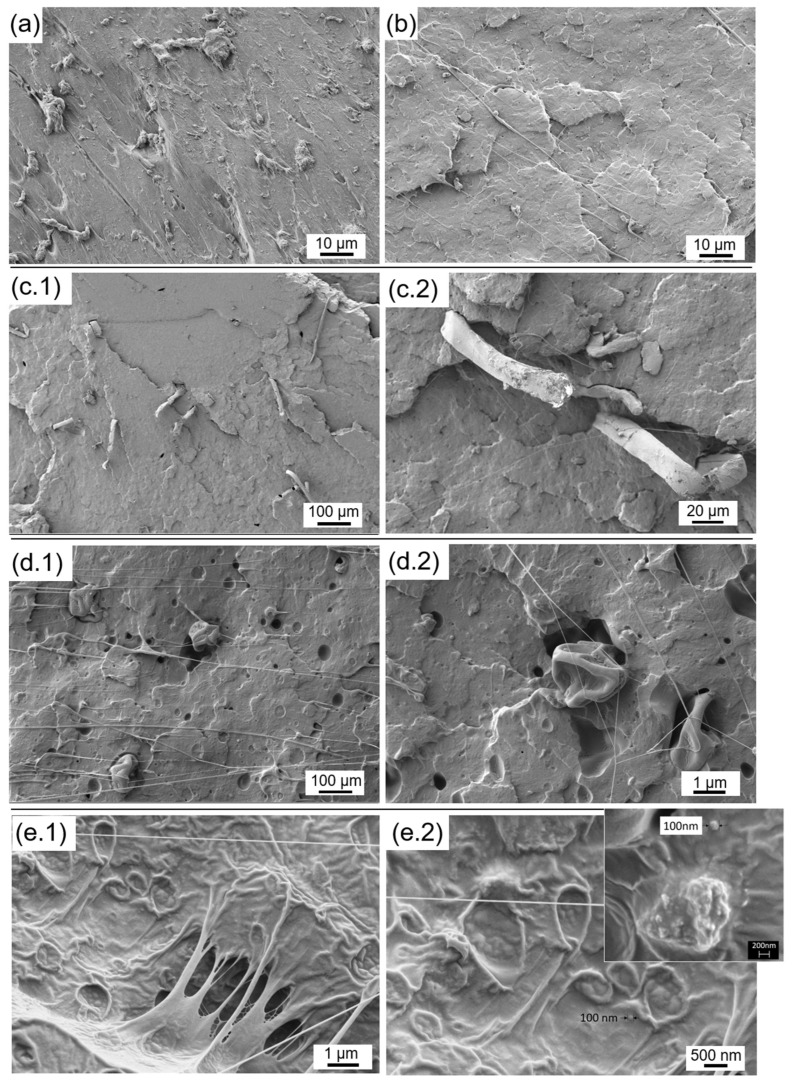
FESEM images of the fracture surface of the PLA-MCO-silk composites: (**a**) Neat PLA, (**b**) PLA-MCO, (**c.1**,**c.2**) PLA-MCO-CS, (**d.1**,**d.2**) PLA-MCO-SFM, and (**e.1**,**e.2**) PLA-MCO-SFN at different magnifications.

FESEM images of the surface of the fractures of the PLA-MCO-silk composites are shown in Figure 8. The image of the neat PLA reveals a smooth surface, suggesting a brittle fracture behavior. However, the PLA with MCO showed flowed threads, probably due to the MCO plasticizer effect. This plasticizing character was observed for all formulations with MCO content at the appropriate magnifications, corroborating the stiffness loss and higher elongation obtained for samples with MCO regarding neat PLA. In addition, it should be noted that the amount of MCO is appropriate since good miscibility is observed since spherical cavities typical of an excess of oil are not observed [9,22]. However, images obtained for the PLA-MCO-CS reveal a good fiber dispersion across the entire section of the sample with adequate cohesion between phases (see Supplementary Material Figure S7). The same uniform dispersion was observed for the SFM microstructure (see Supplementary Material Figure S8). The crushed fibers and SFM were found under the same morphology as obtained. This PLA-MCO matrix acting as a plasticizer, in addition to the macroscopic effect of the solid fragments of the fillers could explain the loss of stiffness observed by nanoindentation and the increase of impact strength when SC and SFM were added. For the SFN formulation, single particles of ~100 nm (see Supplementary Material Figure S6) were found immersed in a highly plasticized matrix. These nanoparticles were homogeneously distributed, aggregated in clusters of ~500 nm or as single nanoparticles, and the macroscopic effect, in addition to the PLA-MCO plasticizer effect, was not produced.

### 3.6. Water Contact Angle Measurements

The water contact angle (*θ*_C_) is a measure of hydrophobicity and/or hydrophilicity of the surface of the samples, with more hydrophilic surfaces having smaller *θ*_C_ values. The contact angles of SF-modified surfaces of samples containing silk fillers as well as the controls are shown in Table 4. On one hand, the incorporation of MCO into the PLA produced a significant increase the in the water contact angle of the samples (*p* < 0.05), agreeing with previous results [8]. On the other hand, the values of the water contact angle of the samples with silk fillers showed two opposite behaviors. While the CS or SFN did not significantly modify the water contact angle of the PLA-MCO, the addition of silk microparticles not only reduced the values of *θ*_C_ found in PLA-MCO (*p* < 0.05) but also in neat PLA (*p* < 0.05).

Previous studies have shown the ability of silk to increase the hydrophilicity of the PLA surfaces, which show lower contact angle values, by enriching the mixtures with nitrogen atoms from the amine (–NH2) and amide groups (–CONH–) of the SF [56,57]. Although, the low percentage in weight of silk fillers in samples (i.e., 0.5 phr) could blur this contribution in the samples with CS or SFN but not in the SFM.

### 3.7. Disintegration under Composting Conditions

**Figure 9:**
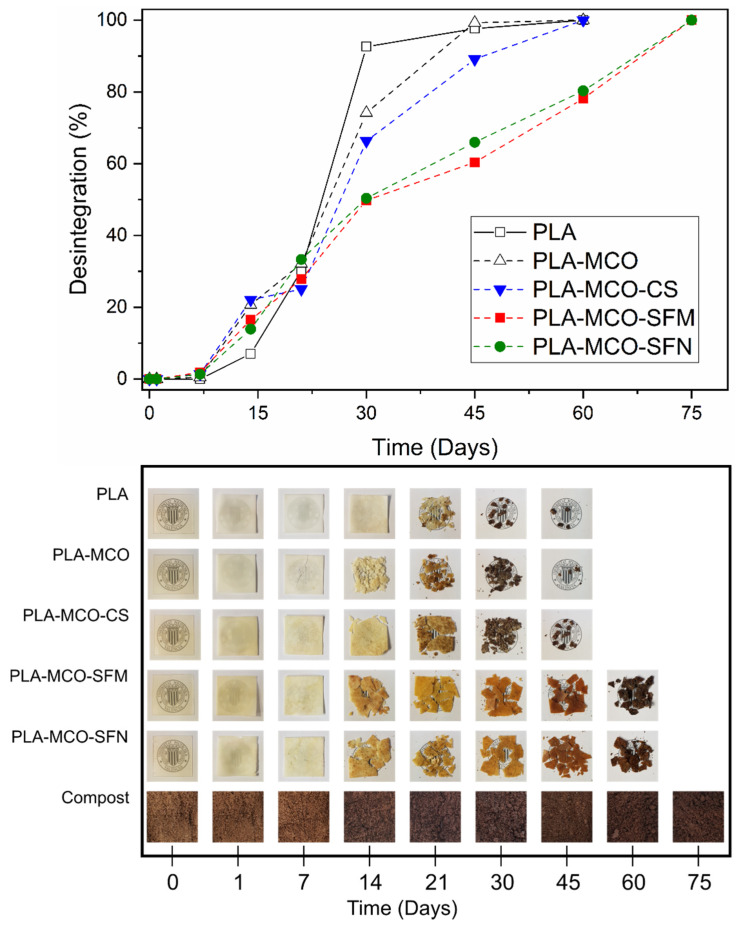
Photographic registry of the Disintegration of PLA and different PLA-composites, containing MCO and silk fillers, under controlled composting conditions.

The disintegration under composting conditions rate and the appearance of the samples on different days of composting is shown in Figure 9. The results for the disintegration of PLA agree with those reported in the literature [58]. In this study, PLA reaches 98% of disintegration in 45 days. A reference value for the disintegration under composting conditions of PLA is a disintegration rate greater than 90% [27]. PLA-MCO samples reached similar disintegration values of PLA.

Results show that the addition of MCO in the formulation does not modify the degree of disintegration of the PLA matrix. The PLA-MCO-CS sample reaches 85% of degradation at day 45. In general, it is observed that the PLA, PLA-MCO, and PLA-MCO-CS (containing crushed silk) samples degrade earlier than the samples that have either micro or nanoparticulated silk fibroin (PLA-MCO-SFM and PLA-MCO-SFN). For these samples, the degree of disintegration is between 60 and 65% in 45 days for PLA-MCO-SFM and PLA-MCO-SFN, respectively. Thus, the presence of micro and nanoparticles has delayed the disintegration of the PLA-MCO matrix under composting conditions.

The results of silk biodegradation in soil agree with previous studies which revealed the strong growth of bacteria on raw silk, but only very limited growth on degummed silk, thus indicating that bacteria more readily use sericin than fibroin as a source of carbon [59,60]. In this regard, the presence of SFM or SFN delayed the composite disintegration by 15 days, agreeing with previous findings.

**Figure 10:**
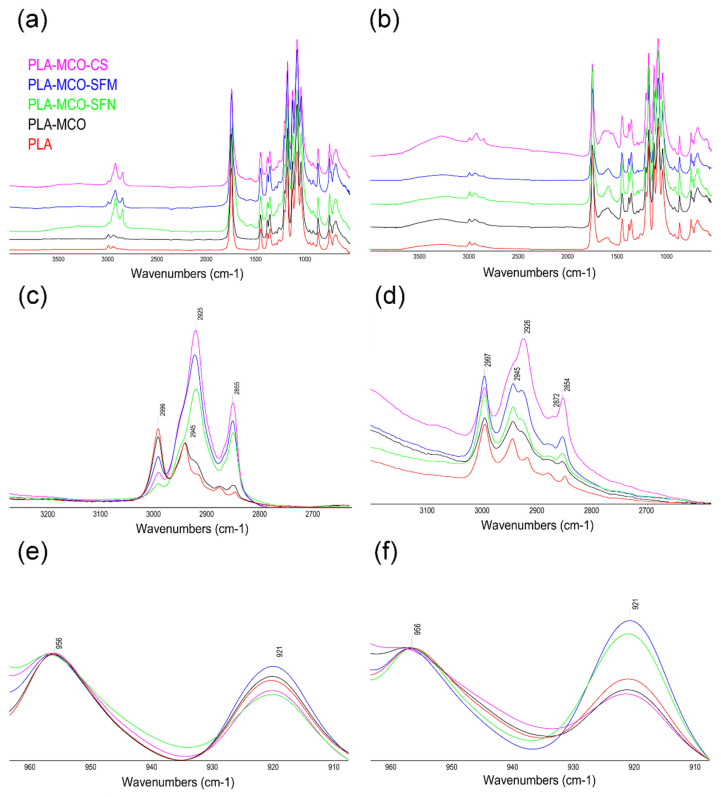
Comparative evolution of the ATR-FTIR spectra along the incubation at composting conditions: Full spectra of the composites at days 1 (**a**) and 30 (**b**). Detail of the spectral region 3200–2700 cm^−1^, at days 1 (**c**) and 30 (**d**). Detail of the spectral region 960–910 cm^−1^, at days 1 (**e**) and 30 (**f**).

These macroscopic results were corroborated by the FTIR analysis of the disintegrated samples. The chemical and structural changes in the materials along the composting period were also followed by ATR-FTIR, and the spectra of analyzed materials under composting conditions (Figure 10) showed important differences concerning those of the starting materials. First, in the spectral region of 3050–2800 cm^−1^, the band at 2996 cm^−1^, assigned to ν_as_(CH_3_), decreased in intensity and the band at 2945 cm^−1^, assigned toν_s_(CH_3_), shifted to 2925 cm^−1^, due the changes in the secondary structure in the composting conditions.

Similarly, in the spectral region of 1800–1700 cm^−1^, corresponding to the C=O stretching, the PLA and PLA-MCO subjected to composting conditions showed a new band region at 1749 cm^−1^. This band appeared to shift to 1746 cm^−1^ for the silk composites. These samples also presented a shoulder at approximately 1700 cm^−1^, which can be assigned to the formation of −COOH groups during hydrolytic degradation [61]. It is also worth noting that, the analysis of the Amide I and II regions of the ATR-FTIR spectra of silk fibroin materials can be used as the most informative for determining their secondary structure [35,62].

**Figure 11:**
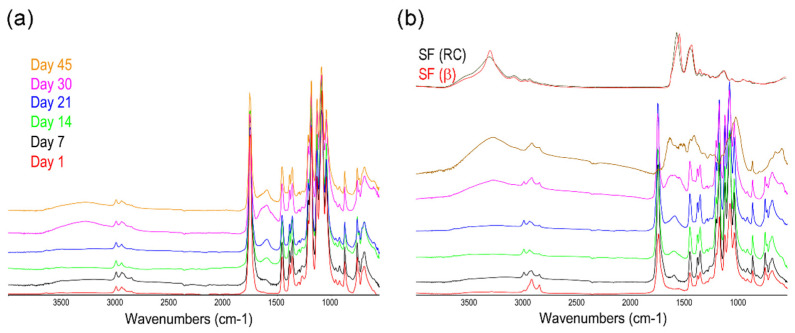
Comparative evolution of the ATR-FTIR spectra along the incubation at composting conditions: Full spectra of the PLA from day 1 to 45 (**a**) and PLA-MCO-CS from day 1 to 45 (**b**). Pure fibroin in random coil (RC) or β-sheet (β) conformation is inserted for comparison purposes.

Figure 11 shows the evolution of the spectra of PLA (a) and PLA-MCO-CS (b) from day 1 to day 45. As can be seen, both silk fibroin Amide I and II bands were hindered by the PLA spectrum due to their low silk/PLA ratio until day 7. From then, a remarkable increase in the signal of the two broad bands centered at 1650 and 1550 cm^−1^ appeared in the PLA-MCO-CS spectra.

Comparatively, the PLA spectra showed an increase in the signal of a broad band centered at 1600 cm^−1^, which is related to the presence of carboxylate ions in degraded PLA [63,64]. The spectra of silk fibroin nanoparticles, mainly in a β-sheet, highly crystalline conformation (amide I at ~1626 cm^−1^ and amide II at ~1520 cm^−1^), and the regenerated silk fibroin (non-crystalline or Silk I), which was mainly in a random coil (RC) conformation (amide I at ~1650 cm^−1^ and amide II at ~1538 cm^−1^), are presented in the figure as a reference. In silk composites, the signals are composed by the sum of the contributions of the random coil and β-sheet conformations. This behavior could be explained by the fact that the decomposition of the PLA facilitates the emergence of the signals corresponding to the crystallized silk fibroin (highly ordered, insoluble, and stable) present in the composites from the background of the PLA spectrum. However, the fibroins have also been partially decomposed during this incubation into soluble peptides, mainly in a “random coil” conformation, giving a combination of both signals in the spectra with wider Amide I and II bands.

## 4. Conclusions

Corn oil was successfully maleinized (MCO) and used together with different silk particles as PLA modifiers. The low intrinsic elongation at break of PLA (1.7%) was improved by adding MCO-CS (23.7%), MCO-SFM (8.8%), and MCO-SFN (10.8%). Field emission scanning microscopy (FESEM) reveals a good miscibility between PLA and MCO and a good dispersion of all the silk particles used. The glass transition temperature is reduced by incorporating the silk particles and the crystallinity increases. Based on UV absorption results, silk particles act as UV light shields in PLA. The results obtained in this work suggest that an environmentally friendly plasticized PLA can be obtained using maleinized corn oil with 0.5 phr silk-based materials crushed silk, silk fibroin microparticles, or nanoparticles). The authors have identified the process of dispersion of the particles in the oil as a limitation of the study since the amount of MCO that can be added to PLA for the optimal plasticizing effect is limited. Consequently, the amount of nano and microparticles that can be homogenously dispersed with this technique is also limited.

Interestingly, these silk-based fillers introduced different mechanical and thermal properties to the composites, which offer a wide range of combinations for customizing the properties by changing the composition of the PLA. Indeed, the successful composting test guarantees the disintegrable nature of the formulations studied and results agree with previous studies.

Finally, further studies focused on obtaining new biocomposites have been carried out, by either testing alternative fillers or new formulation methods, both focused in to overcome the limitations of the study and increase the knowledge in this dynamic field. Among them, the sericin particles are an alternative filler (also a by-product of the silk industry). Another option would be obtaining biodegradable thermoplastics composed of PLA and silk fibers by pultrusion.

## Figures and Tables

**Table 1:** Code and composition of samples PLA-Silk.

Code	PLA (wt.%)	MCO (wt.%)	CS (phr *)	SFM (phr)	SFN (phr)
PLA	100	-	-	-	-
PLA-MCO	90	10	-	-	-
PLA-MCO-CS	90	10	0.5	-	-
PLA-MCO-SFM	90	10	-	0.5	-
PLA-MCO-SFN	90	10	-	-	0.5

* phr (per hundred resin), represents de weight parts of the silk particles added to one hundred weight parts of the base PLA-MCO blend.

**Table 2:** Tensile, flexural, and impact resistance properties of samples PLA-Silk.

Formulation	Tensile Properties		Flexural Properties		Impact Resistance
	Maximum Resistance (MPa)	Elongation at Break (%)	Maximum Resistance (MPa)	Flexural Modulus (MPa)	Charpy’s Impact Energy (kJ·m^2^)
PLA	62.4 ± 1.5 ^a^	1.7 ± 0.9 ^a^	102.3 ± 3.6 ^a^	2609 ± 119 ^a^	29.5 ± 5.5 ^a,b,c^
PLA-MCO	54.7 ± 1.3 ^b^	6.1 ± 2.1 ^a,c^	99.5 ± 2.3 ^a^	2314 ± 251 ^a^	26.4 ± 2.5 ^a,b,c^
PLA-MCO-CS	47.5 ± 1.8 ^c^	23.7 ± 4.7 ^b^	85.2 ± 2.8 ^b^	2652 ±206 ^a^	31.8 ± 5.6 ^b^
PLA-MCO-SFM	48.6 ± 2.6 ^c^	8.8 ± 1.8 ^c^	86.6 ± 3.1 ^b^	2536 ± 98 ^a^	31.3 ± 6.8 ^a,b,c^
PLA-MCO-SFN	52.0 ± 0.9 ^b^	10.8 ± 2.5 ^c^	84.9 ± 3.1 ^b^	2649 ± 147 ^a^	22.6 ± 4.3 ^c^

^a–c^ Different letters within the same property show statistically significant differences between formulations (*p* < 0.05).

**Table 3:** Summary of the DSC and TGA thermal parameters of neat PLA, plasticized PLA, and PLA-MCO-Silk composites.

	DSC 1st Heating						TGA	
Formulation	T_g_ (°C)	T_cc_ (°C)	ΔH_cc_ (J·g^−1^)	T_m_ (°C)	ΔH_m_ (J·g^−1^)	*X_c_* (%)	T_5%_ (°C)	T_max_ (°C)
PLA	62.4	110.1	23.1	148.4	23.6	0.6	305.0	339.6
PLA-MCO	62.3	106.0	17.1	152.0	18.5	1.7	326.3	358.3
PLA-MCO-CS	60.8	103.9	19.5	150.7	20.8	1.5	324.5	361.0
PLA-MCO-SFM	60.9	103.5	21.0	152.4	24.3	3.9	323.0	362.1
PLA-MCO-SFN	60.7	106.3	20.9	150.2	23.0	2.5	322.0	361.0

**Table 4:** Water contact angle values of the samples.

Sample	PLA	PLA-MCO	PLA-MCO-CS	PLA-MCO-SFM	PLA-MCO-SFN
*θ*_C_ (°)	75.0 ± 2.8 ^a^	82.6 ± 1.1 ^b^	81.6 ± 3.1 ^b^	68.7 ± 3.1 ^c^	84.0 ± 2.4 ^b^

Results are expressed as mean ± standard deviation (n = 5). The different lower-case letters (^a–c^) in the same row indicate significantly-different values (*p* < 0.05).

## Data Availability

The data presented in this study are available on request from the corresponding author.

## Supplementary Materials

The following supporting information can be downloaded at: https://www.mdpi.com/article/10.3390/polym14225016/s1, Figure S1. Evolution of the acid value in the different stages of the process of maleinization of corn oil. Figure S2. White silk fibroin cocoons were used for the study under a white light lamp (top) and UV lamp (λ = 365 nm) (down). Figure S3. Micrographs of the silk fillers under white light and UV light and fluorescence filters. Figure S4. SEM micrograph of the crushed silk fibers. Figure S5. SEM micrograph of the silk microparticles. Figure S6. SEM micrograph of the silk nanoparticles. Figure S7. SEM micrograph of a silk fiber included in the PLA-MCO matrix. Figure S8. SEM micrograph of silk microparticles included in the PLA-MCO matrix. Figure S9. Micrographs used for the measurement of the water contact angle (θw) of the samples: (a) PLA, (b) PLA-MCO, (c) PLA-MCO-CS, (d) PLA-MCO-SFM and (e) PLA-MCO-SFN. Values are inserted. Figure S10. Micrographs of the samples stained with Coomassie blue. (Scale bar: 500 µm (5×) or 100 µm (10×)) [32,65,66,67].

## References

[1] Zhou P., Li C., Bai Y., Dong S., Xian G., Vedernikov A., Akhatov I., Safonov A., Yue Q. (2022). Durability study on the interlaminar shear behavior of glass-fibre reinforced polypropylene (GFRPP) bars for marine applications. Constr. Build. Mater..

[2] Minchenkov K., Vedernikov A., Kuzminova Y., Gusev S., Sulimov A., Gulyaev A., Kreslavskaya A., Prosyanoy I., Xian G., Akhatov I. (2022). Effects of the quality of pre-consolidated materials on the mechanical properties and morphology of thermoplastic pultruded flat laminates. Compos. Commun..

[3] Vedernikov A., Minchenkov K., Gusev S., Sulimov A., Zhou P., Li C., Xian G., Akhatov I., Safonov A. (2022). Effects of the Pre-Consolidated Materials Manufacturing Method on the Mechanical Properties of Pultruded Thermoplastic Composites. Polymers.

[4] Bioplastics Market Development Update 2021. European Bioplastics Association. Berlin, Germany. https://docs.european-bioplastics.org/publications/market_data/Report_Bioplastics_Market_Data_2021_short_version.pdf.

[5] Lomwongsopon P., Varrone C. (2022). Contribution of Fermentation Technology to Building Blocks for Renewable Plastics. Fermentation.

[6] Luzi F., Dominici F., Armentano I., Fortunati E., Burgos N., Fiori S., Jiménez A., Kenny J.M., Torre L. (2019). Combined effect of cellulose nanocrystals, carvacrol and oligomeric lactic acid in PLA_PHB polymeric films. Carbohydr. Polym..

[7] Balart J.F., Montanes N., Fombuena V., Boronat T., Sánchez-Nacher L. (2017). Disintegration in Compost Conditions and Water Uptake of Green Composites from Poly(Lactic Acid) and Hazelnut Shell Flour. J. Polym. Environ..

[8] Garcia-Garcia D., Carbonell-Verdu A., Arrieta M.P., López-Martínez J., Samper M. (2020). Improvement of PLA film ductility by plasticization with epoxidized karanja oil. Polym. Degrad. Stab..

[9] Ferri J.M., Samper M.D., García-Sanoguera D., Reig M.J., Fenollar O., Balart R. (2016). Plasticizing effect of biobased epoxidized fatty acid esters on mechanical and thermal properties of poly(lactic acid). J. Mater. Sci..

[10] Borysiuk P., Boruszewski P., Auriga R., Danecki L., Auriga A., Rybak K., Nowacka M. (2021). Influence of a bark-filler on the properties of PLA biocomposites. J. Mater. Sci..

[11] Arrieta M.P., Samper M.D., Aldas M., López J. (2017). On the Use of PLA-PHB Blends for Sustainable Food Packaging Applications. Materials.

[12] Su S., Kopitzky R., Tolga S., Kabasci S. (2019). Polylactide (PLA) and Its Blends with Poly(butylene succinate) (PBS): A Brief Review. Polymers.

[13] Zeng J.-B., Li K.-A., Du A.-K. (2015). Compatibilization strategies in poly(lactic acid)-based blends. RSC Adv..

[14] Bocqué M., Voirin C., Lapinte V., Caillol S., Robin J.-J. (2015). Petro-based and bio-based plasticizers: Chemical structures to plasticizing properties. J. Polym. Sci. Part A Polym. Chem..

[15] Omar A.A., Hanafi M.H.M., Razak N.H., Ibrahim A., Razak N.A.A. (2021). A Best-Evidence Review of Bio-Based Plasticizer and the Effects on the Mechanical Properties of PLA. Chem. Eng. Trans..

[16] Beltrán F.R., Gaspar G., Chomachayi M.D., Jalali-Arani A., Lozano-Pérez A.A., Cenis J.L., de la Orden M.U., Pérez E., Urreaga J.M.M. (2020). Influence of addition of organic fillers on the properties of mechanically recycled PLA. Environ. Sci. Pollut. Res..

[17] Aworinde A.K., Adeosun S.O., Oyawale F.A., Akinlabi E.T., Akinlabi S. (2020). Comparative effects of organic and inorganic bio-fillers on the hydrophobicity of polylactic acid. Results Eng..

[18] Scaffaro R., Maio A., Gulino E., Alaimo G., Morreale M. (2021). Green Composites Based on PLA and Agricultural or Marine Waste Prepared by FDM. Polymers.

[19] Al-Mulla E.A.J., Ibrahim N.A.B., Shameli K., Bin Ahmad M., Yunus W.M.Z.W. (2013). Effect of epoxidized palm oil on the mechanical and morphological properties of a PLA–PCL blend. Res. Chem. Intermed..

[20] Ferri J.M., Garcia-Garcia D., Montanes N., Fenollar O., Balart R. (2017). The effect of maleinized linseed oil as biobased plasticizer in poly(lactic acid)-based formulations. Polym. Int..

[21] Carbonell-Verdu A., Garcia-Garcia D., Dominici F., Torre L., Sanchez-Nacher L., Balart R. (2017). PLA films with improved flexibility properties by using maleinized cottonseed oil. Eur. Polym. J..

[22] Carbonell-Verdu A., Samper M.D., Garcia-Garcia D., Sanchez-Nacher L., Balart R. (2017). Plasticization effect of epoxidized cottonseed oil (ECSO) on poly(lactic acid). Ind. Crop. Prod..

[23] Quiles-Carrillo L., Montanes N., Lagaron J.M., Balart R., Torres-Giner S. (2018). On the use of acrylated epoxidized soybean oil as a reactive compatibilizer in injection-molded compostable pieces consisting of polylactide filled with orange peel flour. Polym. Int..

[24] Thakur S., Cisneros-Lopez E.O., Pin J.-M., Misra M., Mohanty A.K. (2019). Green Toughness Modifier from Downstream Corn Oil in Improving Poly(lactic acid) Performance. ACS Appl. Polym. Mater..

[25] Payne A.R. (1965). Effect of dispersion on the dynamic properties of filler-loaded rubbers. J. Appl. Polym. Sci..

[26] Chomachayi M.D., Jalali-Arani A., Urreaga J.M. (2021). A Comparison of the Effect of Silk Fibroin Nanoparticles and Microfibers on the Reprocessing and Biodegradability of PLA/PCL Blends. J. Polym. Environ..

[27] Beltrán F., Arrieta M., Antón D.E., Lozano-Pérez A., Cenis J., Gaspar G., de la Orden M., Urreaga J.M. (2021). Effect of Yerba Mate and Silk Fibroin Nanoparticles on the Migration Properties in Ethanolic Food Simulants and Composting Disintegrability of Recycled PLA Nanocomposites. Polymers.

[28] Omenetto F.G., Kaplan D.L. (2010). New Opportunities for an Ancient Material. Science.

[29] Rockwood D.N., Preda R.C., Yücel T., Wang X., Lovett M.L., Kaplan D.L. (2011). Materials fabrication from Bombyx mori silk fibroin. Nat. Protoc..

[30] Craig C.L., Riekel C. (2002). Comparative architecture of silks, fibrous proteins and their encoding genes in insects and spiders. Comp. Biochem. Physiol. Part B Biochem. Mol. Biol..

[31] Altman G.H., Diaz F., Jakuba C., Calabro T., Horan R.L., Chen J., Lu H., Richmond J., Kaplan D.L. (2003). Silk-based biomaterials. Biomaterials.

[32] Perez-Nakai A., Lerma-Canto A., Domingez-Candela I., Garcia-Garcia D., Ferri J., Fombuena V. (2021). Comparative Study of the Properties of Plasticized Polylactic Acid with Maleinized Hemp Seed Oil and a Novel Maleinized Brazil Nut Seed Oil. Polymers.

[33] Sempere-Torregrosa J., Ferri J.M., de la Rosa-Ramírez H., Pavon C., Samper M.D. (2022). Effect of Epoxidized and Maleinized Corn Oil on Properties of Polylactic Acid (PLA) and Polyhydroxybutyrate (PHB) Blend. Polymers.

[34] Rodriguez-Nogales A., Lozano-Pérez A., Aznar-Cervantes S., Algieri F., Garrido-Mesa J., Vezza T., Utrilla M., Cenis J., Rodríguez-Cabezas M., Gálvez J. (2016). Effect of aqueous and particulate silk fibroin in a rat model of experimental colitis. Int. J. Pharm..

[35] Carissimi G., Lozano-Pérez A.A., Montalbán M.G., Aznar-Cervantes S.D., Cenis J.L., Víllora G. (2019). Revealing the Influence of the Degumming Process in the Properties of Silk Fibroin Nanoparticles. Polymers.

[36] Ajisawa A. (1998). Dissolution of Silk Fibroin with Calcium Chloride/Ethanol Aqueous Solution. J. Sericultural Sci. Jpn..

[37] (2019). Determination of Tensile Properties—Part 1: General Principles.

[38] (2010). Plastics—Determination of Charpy Impact Properties Part 1: Non-Instrumented Impact Test.

[39] (2018). Plastics—Determination of Tensile Properties—Test Conditions for Films and Sheets.

[40] (2013). International Standards Organization Plastics-Determination of Flexural Properties.

[41] (2020). Plastics—Determination of Charpy Impact Properties—Part 2: Instrumented Impact Test.

[42] (2015). International Organization for Standardization Plastics—Determination of the Degree of Disintegration of Plastic Materials under Simulated Composting Conditions in a Laboratory-Scale Test.

[43] Arrieta M.P., Castro-Lopez M.D.M., Rayón E., Barral-Losada L.F., López-Vilariño J.M., López J., González-Rodríguez M.V. (2014). Plasticized Poly(lactic acid)–Poly(hydroxybutyrate) (PLA–PHB) Blends Incorporated with Catechin Intended for Active Food-Packaging Applications. J. Agric. Food Chem..

[44] Rayón E., Arrieta M., Pasíes T., López J., Jordá J. (2018). Enhancing the mechanical features of clay surfaces by the absorption of nano-SiO_2_ particles in aqueous media. Case of study on Bronze Age clay objects. Cem. Concr. Compos..

[45] Ferri J.M., Garcia-Garcia D., Sánchez-Nacher L., Fenollar O., Balart R. (2016). The effect of maleinized linseed oil (MLO) on mechanical performance of poly(lactic acid)-thermoplastic starch (PLA-TPS) blends. Carbohydr. Polym..

[46] Deng Q., Wang F., Gough C.R., Hu X. (2022). Tunable microphase-regulated silk fibroin/poly (lactic acid) biocomposite materials generated from ionic liquids. Int. J. Biol. Macromol..

[47] Dominguez-Candela I., Ferri J.M., Cardona S.C., Lora J., Fombuena V. (2021). Dual Plasticizer/Thermal Stabilizer Effect of Epoxidized Chia Seed Oil (*Salvia hispanica* L.) to Improve Ductility and Thermal Properties of Poly(Lactic Acid). Polymers.

[48] Chen X., Han L., Zhang T., Zhang J. (2014). Influence of crystal polymorphism on crystallinity calculation of poly(l-lactic acid) by infrared spectroscopy. Vib. Spectrosc..

[49] Zhang J., Duan Y., Sato H., Tsuji H., Noda I., Yan S., Ozaki Y. (2005). Crystal Modifications and Thermal Behavior of Poly(l-lactic acid) Revealed by Infrared Spectroscopy. Macromolecules.

[50] Phan Q.T., Le M.H., Le T.T.H., Tran T.H.H., Xuan P.N., Ha P.T. (2016). Characteristics and cytotoxicity of folate-modified curcumin-loaded PLA-PEG micellar nano systems with various PLA:PEG ratios. Int. J. Pharm..

[51] Cele H., Ojijo V., Chen H., Kumar S., Land K., Joubert T., de Villiers M., Ray S. (2014). Effect of nanoclay on optical properties of PLA/clay composite films. Polym. Test..

[52] Breslauer D.N., Kaplan D.L. (2012). Silks: Properties and uses of natural and designed variants. Biopolymers.

[53] Nanda M.R., Misra M., Mohanty A.K. (2011). The Effects of Process Engineering on the Performance of PLA and PHBV Blends. Macromol. Mater. Eng..

[54] Wang S., Zhang Y., Ren W., Zhang Y., Lin H. (2005). Morphology, mechanical and optical properties of transparent BR/clay nanocomposites. Polym. Test..

[55] Sionkowska A., Planecka A. (2011). The influence of UV radiation on silk fibroin. Polym. Degrad. Stab..

[56] Cai K., Yao K., Lin S., Yang Z., Li X., Xie H., Qing T., Gao L. (2001). Poly(d,l-lactic acid) surfaces modified by silk fibroin: Effects on the culture of osteoblast in vitro. Biomaterials.

[57] Cai K., Yao K., Cui Y., Yang Z., Li X., Xie H., Qing T., Gao L. (2001). Influence of different surface modification treatments on poly(d,l-lactic acid) with silk fibroin and their effects on the culture of osteoblast in vitro. Biomaterials.

[58] Tokiwa Y., Calabia B.P. (2006). Biodegradability and biodegradation of poly(lactide). Appl. Microbiol. Biotechnol..

[59] Seves A., Romanò M., Maifreni T., Sora S., Ciferri O. (1998). The microbial degradation of silk: A laboratory investigation. Int. Biodeterior. Biodegrad..

[60] Szostak-Kotowa J. (2004). Biodeterioration of textiles. Int. Biodeterior. Biodegrad..

[61] Auras R., Lim L.T., Selke S.E.M., Tsuji H., Auras R., Lim L.-T., Selke S.E.M., Tsuji H. (2010). Poly(Lactic Acid).

[62] Hu X., Kaplan A.D., Cebe P. (2006). Determining Beta-Sheet Crystallinity in Fibrous Proteins by Thermal Analysis and Infrared Spectroscopy. Macromolecules.

[63] Arrieta M., Fortunati E., Dominici F., Rayón E., López J., Kenny J. (2014). PLA-PHB/cellulose based films: Mechanical, barrier and disintegration properties. Polym. Degrad. Stab..

[64] Fortunati E., Armentano I., Iannoni A., Barbale M., Zaccheo S., Scavone M., Visai L., Kenny J.M. (2011). New multifunctional poly(lactide acid) composites: Mechanical, antibacterial, and degradation properties. J. Appl. Polym. Sci..

[65] (2010). Animal and Vegetable Fats and Oils—Determination of Acid Value and Acidity.

[66] Quiles-Carrillo L., Montanes N., Sammon C., Balart R., Torres-Giner S. (2018). Compatibilization of highly sustainable polylactide/almond shell flour composites by reactive extrusion with maleinized linseed oil. Ind. Crops Prod..

[67] Laemmli U.K. (1970). Cleavage of structural proteins during the assembly of the head of bacteriophage T4. Nature.

