# Supplementary material for: The Influence of Different Sustainable Silk-Based Fillers on the Thermal and Mechanical Properties of Polylactic Acid Composites
Source: Polymers (Basel). 2022 Nov 18;14(22):5016. doi: 10.3390/polym14225016 (PMC9695667; doi:10.3390/polym14225016)
Supplement: Supplementary file 1 [file polymers-14-05016-s001.zip › polymers-2049779-supplementary.pdf]

Supplementary Material for

Influence of different silk-based fillers on the thermal and mechanical properties of polylactic acid composites

José Miguel Ferri[1], Miguel Aldas[1,2], Emilio Rayon[1], Mª Dolores Samper[1*], Antonio Abel Lozano-Pérez[3,4]

[1] *Instituto de Tecnología de Materiales (ITM)-Universitat Politècnica de València (UPV) Plaza Ferrándiz y Carbonell 1, 03801, Alcoy, Alicante, Spain*

[2] *Departamento de Ciencia de Alimentos y Biotecnología, Facultad de Ingeniería Química y Agroindustria, Escuela Politécnica Nacional, 170517, Quito, Ecuador*

[3] *Departamento de Biotecnología, Genómica y Mejora Vegetal, Instituto Murciano de Investigación y Desarrollo Agrario y Medioambiental (IMIDA), 30150, La Alberca (Murcia), Spain*

[4] *Instituto Murciano de Investigación Biosanitaria (IMIB)-Arrixaca, 30120, Murcia, Spain*

* Corresponding author: A.A. Lozano-Pérez

E-mail: abel@um.es

Tel.: +34-968-36-85-86

List of Figures

Figure S1. Evolution of the acid value in the different stages of the process of maleinization of corn oil.

Figure S2. White silk fibroin cocoons used for the study under white light lamp (top) and UV lamp (λ=365nm) (down).

Figure S3. Micrographs of the silk fillers under white light and UV light and fluorescence filters.

Figure S4. SEM micrograph of the crushed silk fibers.

Figure S5. SEM micrograph of the silk microparticles.

Figure S6. SEM micrograph of the silk nanoparticles.

Figure S7. SEM micrograph of a silk fiber included in the PLA-MCO matrix.

Figure S8. SEM micrograph of silk microparticles included in the PLA-MCO matrix.

Figure S9. Micrographs used for the measurement of the water contact angle (θw) of the samples: (a) PLA, (b) PLA-MCO, (c) PLA-MCO-CS, (d) PLA-MCO-SFM and e) PLA-MCO-SFN. Values are inserted.

Figure S10. Micrographs of the samples stained with Coomassie blue. (Scale bar: 500 µm (5×) or 100 µm (10×)).

References

S1. Determination of the degree of maleinization

The degree of maleinization was determined following the guidelines set by the ISO 660:2009 standard [1] by using the following expression:

$$Acid\ Value = \frac{56.1 \times c \times V}{m} \quad (1)$$

Where c is the exact concentration of the KOH standard solution used (mol·L^{-1}), V is the volume of the KOH standard solution used (expressed in mL) and m the analyzed mass (g). Figure 2 shows the evolution of the acid number values at the end of each of the stages at different temperatures, 180, 200 and 220 ºC. Initially the AV is 0.15 mg KOH g^{-1} and at the end of the first hour, at 180 ºC, it is observed that it increases to 24.4 mg KOH g^{-1}, verifying that the maleinization reaction is occurring. After the second hour, at 200 °C, another large increase is observed until reaching an AV value of 87.8 mg KOH g^{-1} and, finally, after the last stage, at 220 °C, the AV reaches 109.2 mg KOH g^{-1}. These results are in total agreement with the values obtained by A. Perez-Nakai *et al.* With an AV of 105 for the maleinized hemp seed and 130 for the maleinized brazil nut at the end of the epoxidation process [2]. Furthermore, Quiles Carrillo *et al.* indicate that commercial grade maleinized linseed oil has a VA of between 105-130 mg KOH g^{-1} [3].

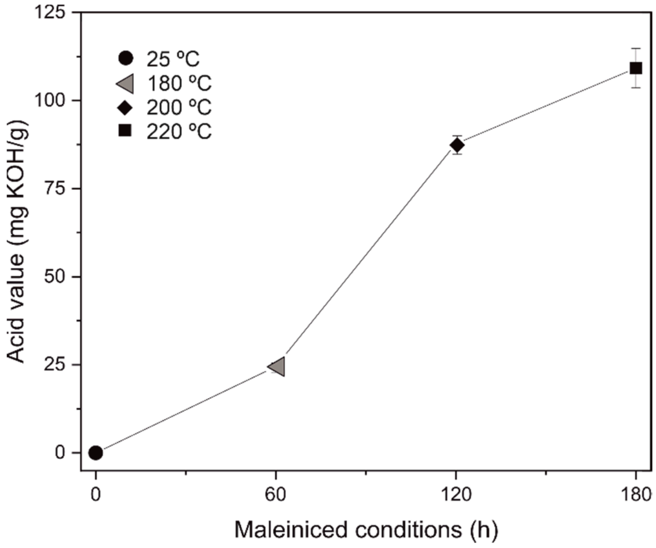

Figure S1. Evolution of the acid value in the different stages of the process of maleinization of corn oil.

S2. Silk Fillers

S2.1 Characterization.

Microscopic analysis of the samples was also performed by using a Nikon Eclipse 50*i* microscope (Nikon Instrument Inc., Melville, NY, USA) equipped with Plan Fluor objectives (5×, 10×, 20×, 40×, 50× and 100×), fluorescence filters for DAPI (UV-2E/C), FITC (B-2E/C) and TRITC (G-2E/C), and a 100 W mercury lamp as light source. Images were captured by a Nikon DS-Fi1-U2 camera controlled by NIS-Elements F software (Ver. 3.00, SP7, Build 547).

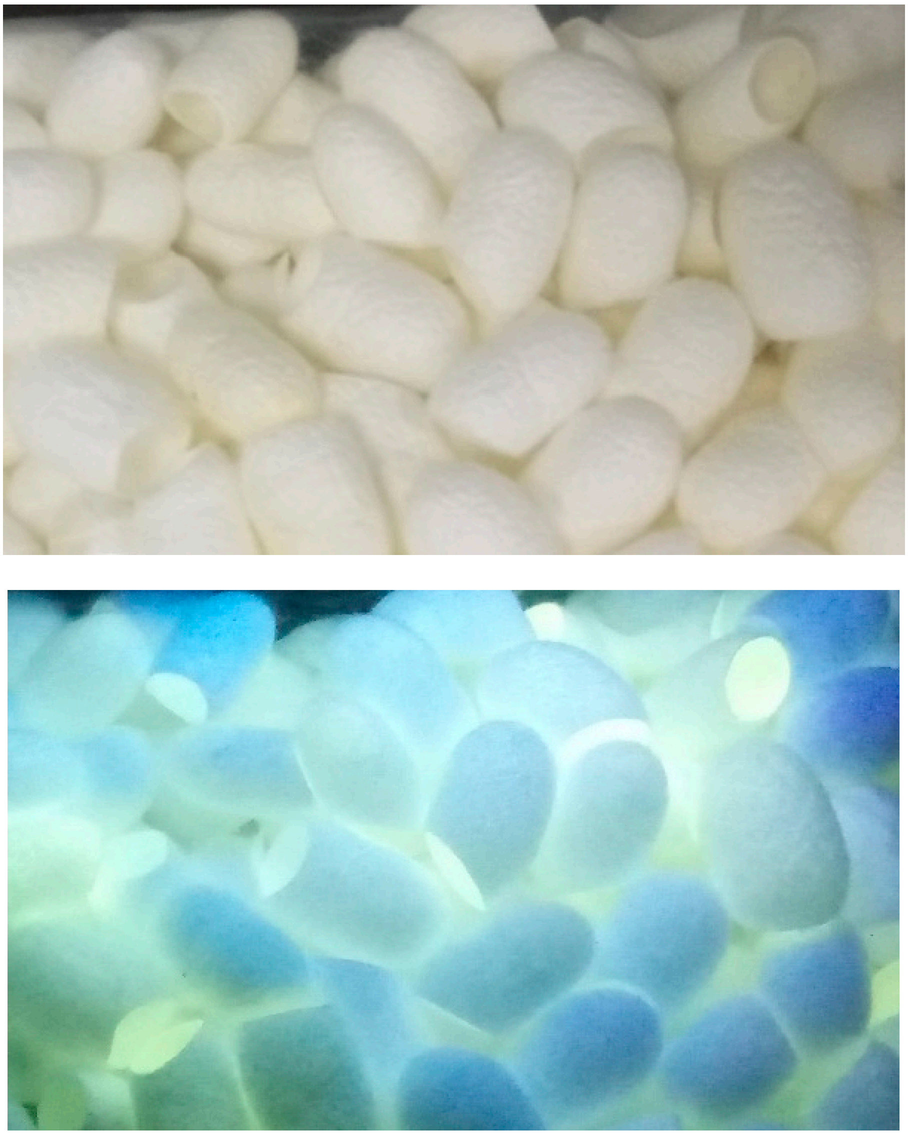

Figure S2. White silk fibroin cocoons used for the study under white light lamp (top) and UV lamp (λ=365nm) (down).

ADDITIVE	Bright field	UV+DAPI Filter	UV+FITC Filter
-Raw cocoon<br><br>(Scale bar: 500µm)	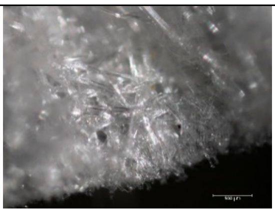	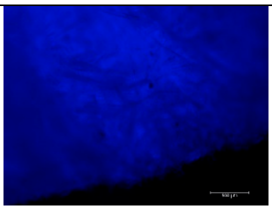	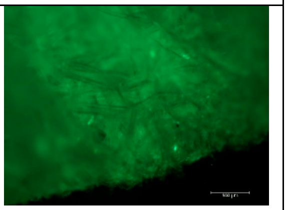
CS<br><br>(Scale bar: 100 µm)	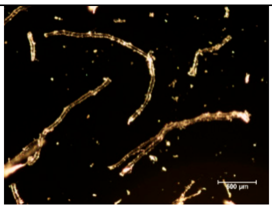	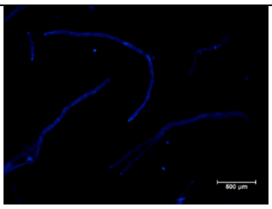	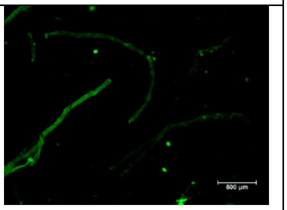
SFM<br><br>(Scale bar: 100 µm)	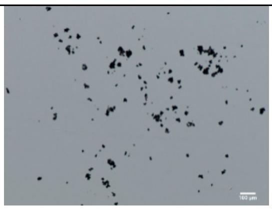	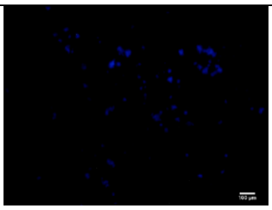	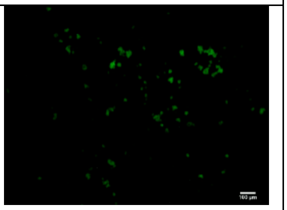
SFN<br><br>(Scale bar: 500 µm)	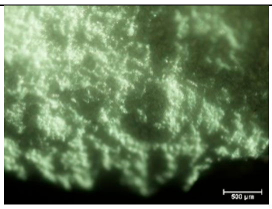	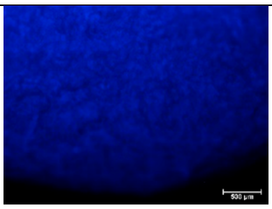	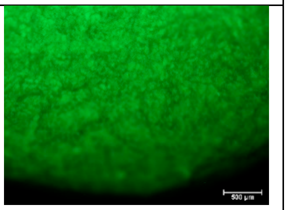

Figure S3. Micrographs of the silk fillers under white light and UV light and fluorescence filters.

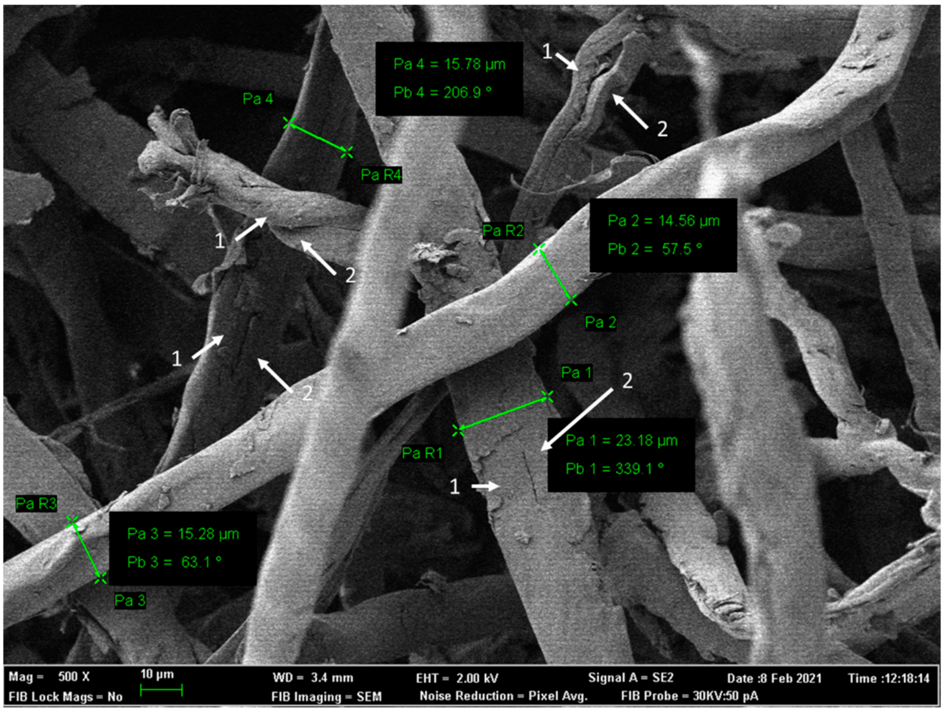

Figure S4. SEM micrograph of the crushed silk fibres.

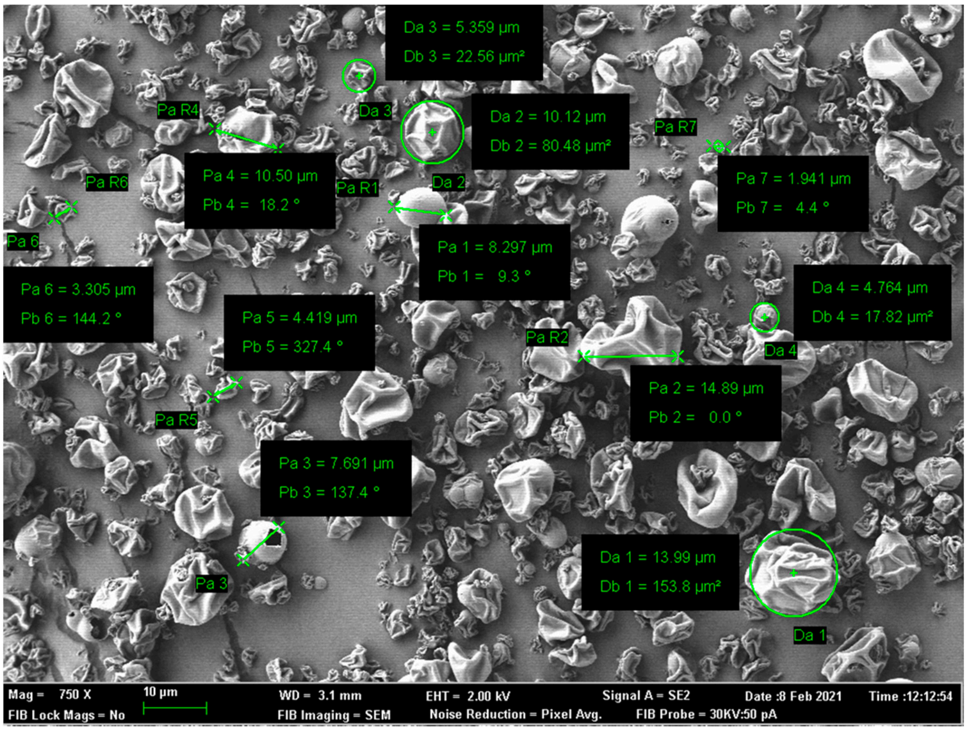

Figure S5. SEM micrograph of the silk microparticles.

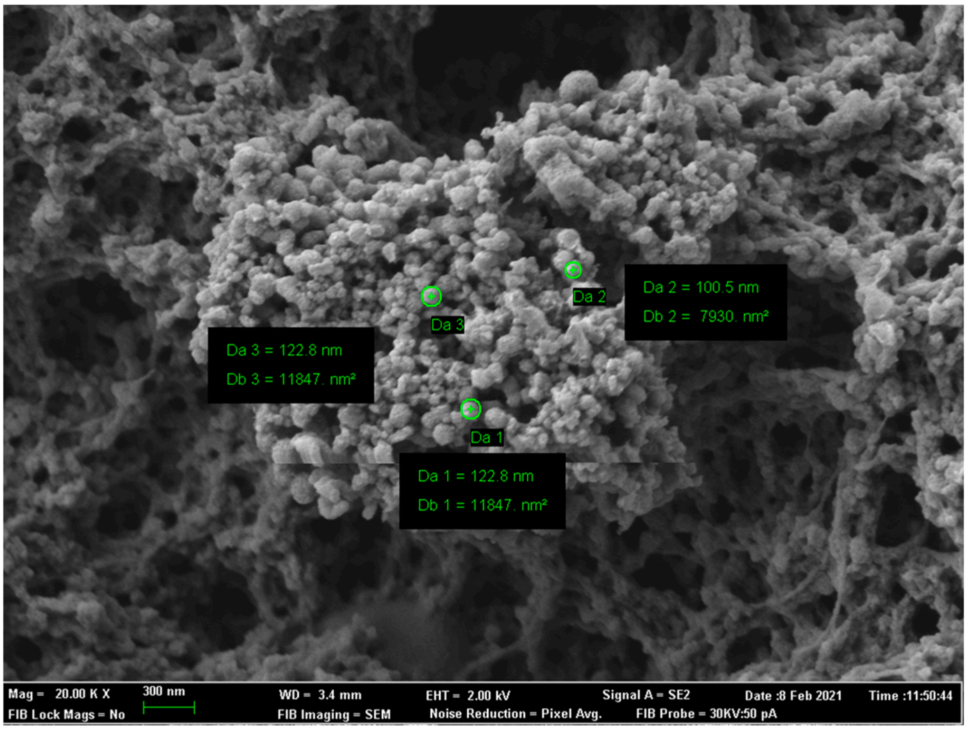

Figure S6. SEM micrograph of the silk nanoparticles.

S3. Characterization of PLA-MCO-silk composites

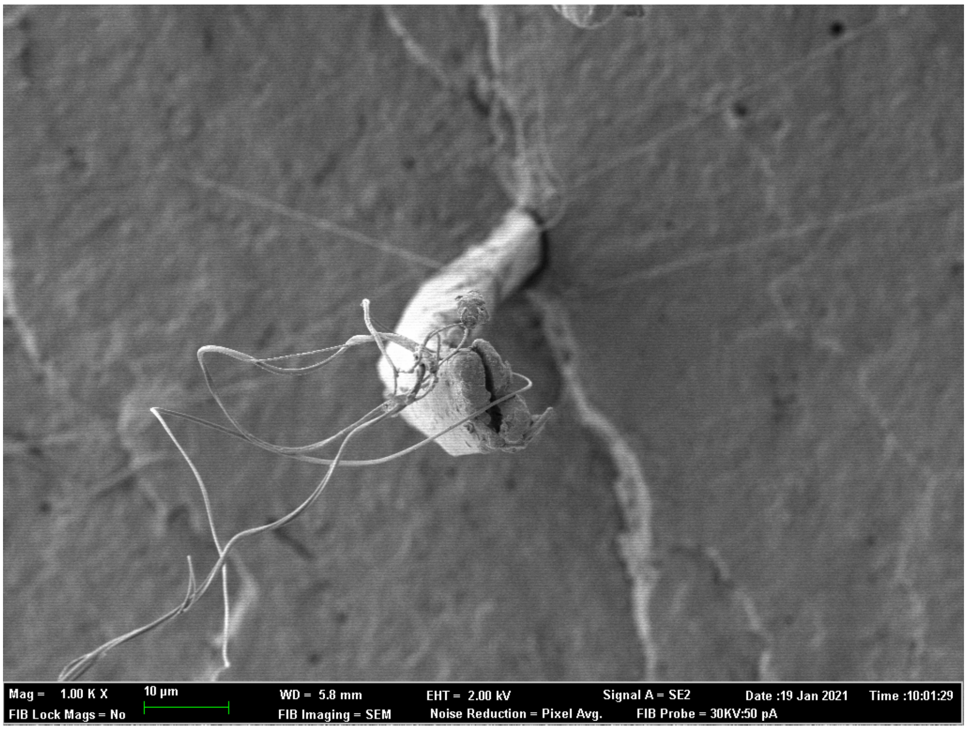

Figure S7. SEM micrograph of a silk fiber included in the PLA-MCO matrix.

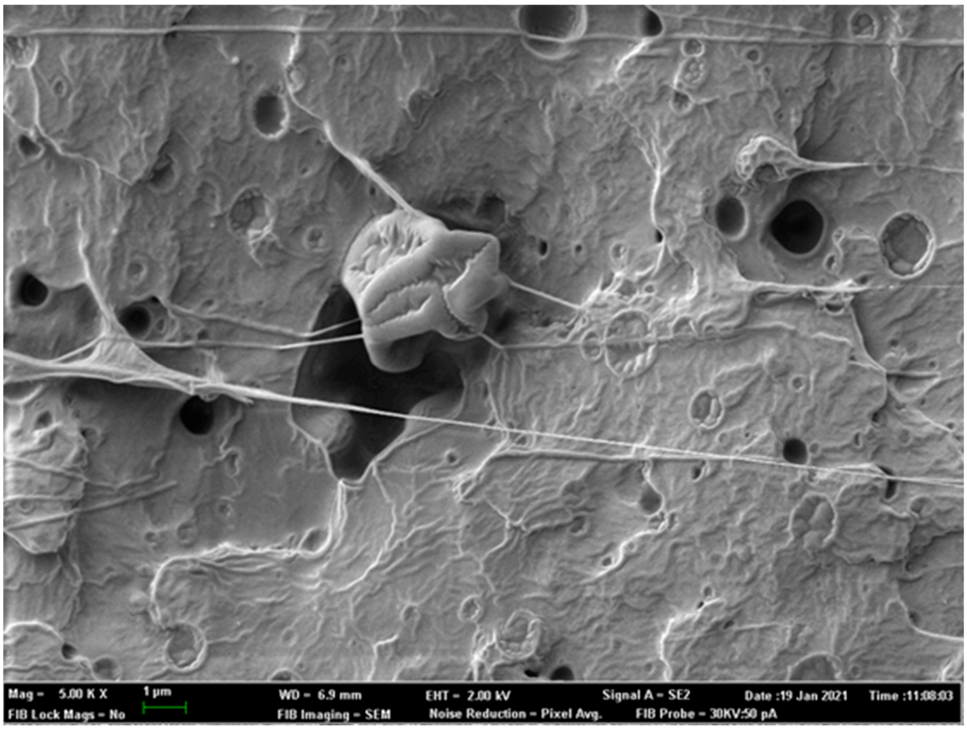

Figure S8. SEM micrograph of silk microparticles included in the PLA-MCO matrix.

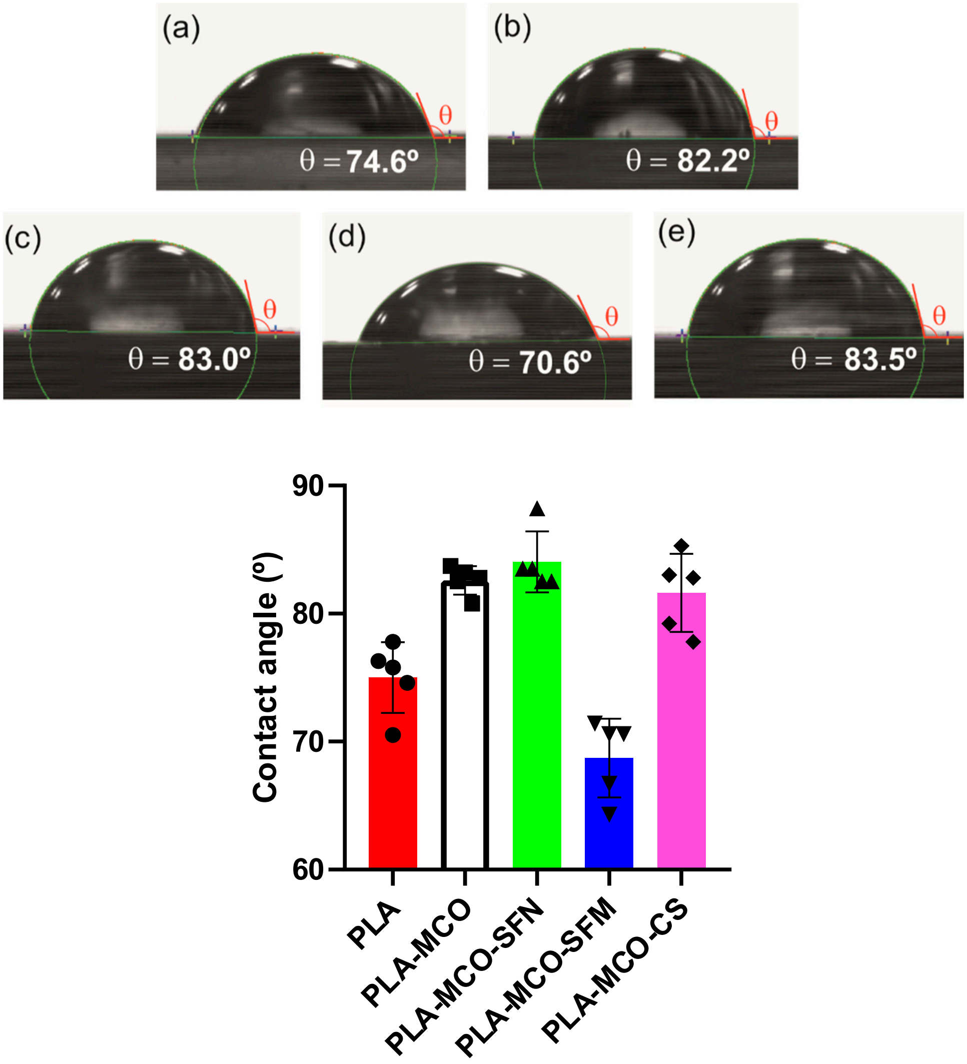

Figure S9. Micrographs used for the measurement of the water contact angle (θw) of the samples: (a) PLA, (b) PLA-MCO, (c) PLA-MCO-CS, (d) PLA-MCO-SFM and e) PLA-MCO-SFN. Values are inserted.

Fractured samples were stained with 0.25% Coomassie Brilliant Blue (Acros Organics, Geel, Belgium), fixed and discolored in ethanol/water mixture, following the stablished protocol for staining proteins in the electrophoresis polyacrylamide gels as described by Laemli *et al.* [4] and photographed for analysis.

Sample	General view	Detail
-PLA-MCO	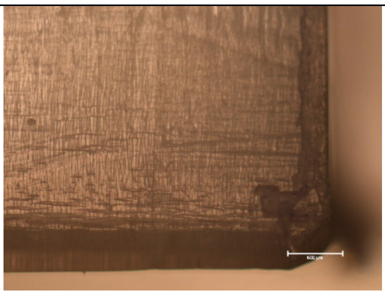	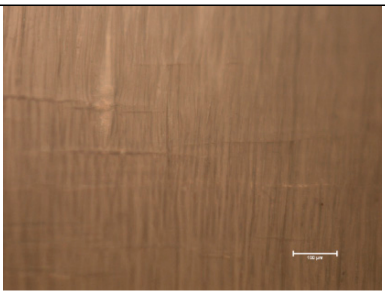
PLA-MCO-CS	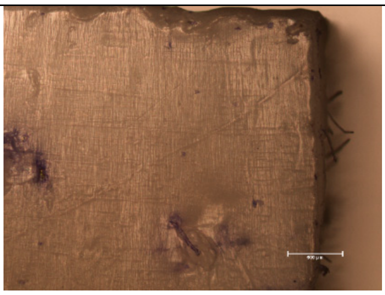	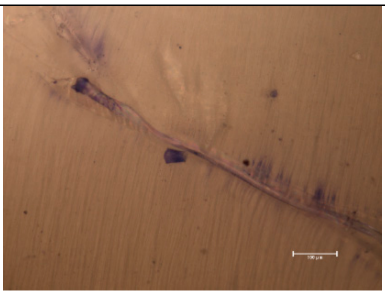
PLA-MCO-SFM	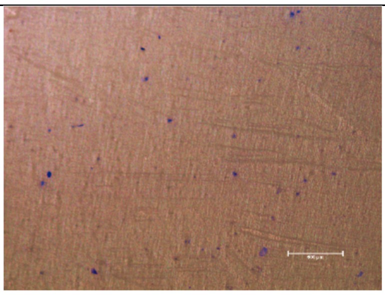	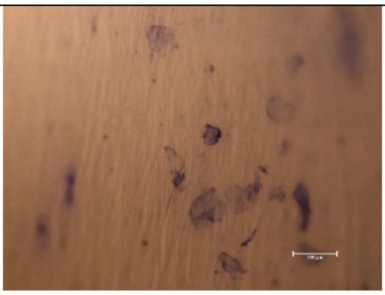
PLA-MCO-SFN	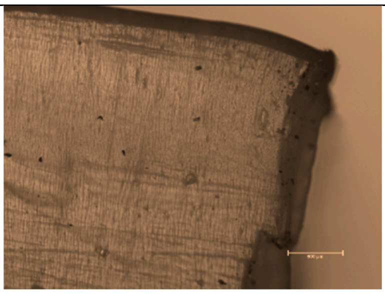	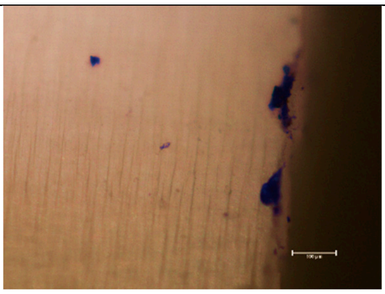

Figure S10. Micrographs of the samples stained with Coomassie blue. (Scale bar: 500 µm (5×) or 100 µm (10×)).

References

1. International Standard Organization Animal and vegetable fats and oils - Determination of acid value and acidity (ISO 660:2009). *Int. Stand.* **2010**, *3*.

2. Perez-Nakai, A.; Lerma-Canto, A.; Domingez-Candela, I.; Garcia-Garcia, D.; Ferri, J.M.; Fombuena, V. Comparative study of the properties of plasticized polylactic acid with maleinized hemp seed oil and a novel maleinized brazil nut seed oil. *Polymers (Basel).* **2021**, *13*.

3. Quiles-Carrillo, L.; Montanes, N.; Sammon, C.; Balart, R.; Torres-Giner, S. Compatibilization of highly sustainable polylactide/almond shell flour composites by reactive extrusion with maleinized linseed oil. *Ind. Crops Prod.* **2018**, *111*.

4. Laemmli, U.K. Cleavage of structural proteins during the assembly of the head of bacteriophage T4. *Nature* **1970**.
